# Primary care in supplementary health: assessment of costs in the care of older adult patients with heart diseases

**DOI:** 10.1590/0034-7167-2022-0486

**Published:** 2023-07-10

**Authors:** Geórgia Silva Marques, Alessandra Maciel Almeida, Isabel Cristina Gomes, Michele Renata Barbosa da Silva, Bruno Almeida Rezende

**Affiliations:** I Faculdade de Ciências Médicas. Belo Horizonte, Minas Gerais, Brazil; II Fundação São Francisco Xavier. Ipatinga, Minas Gerais, Brazil

**Keywords:** Aged, Cardiovascular Diseases, Costs and Cost Analysis, Primary Health Care, Learning Health System, Anciano, Enfermedades Cardiovasculares, Costos y Análisis de Costo, Atención Primaria de Salud, Modelos de Atención de Salud, Idoso, Doenças Cardiovasculares, Custos e Análise de Custo, Atenção Primária à Saúde, Modelos de Assistência à Saúde

## Abstract

**Objectives:**

to identify whether implementing a supplementary Primary Health Care (PHC) system makes it possible to reduce care costs for older adults with heart diseases.

**Methods:**

a retrospective cohort of 223 patients with heart disease aged ≥ 60 years. Data were obtained from medical records and cost databases, assessed for a period of one year before and after PHC implementation. The results were expressed as mean absolute frequencies for number of hospitalizations and as average annual expenses expressed in dollars (US$) in relation to cost data.

**Results:**

there was a reduction in hospitalization expenses after implementing supplementary PHC (p=0.01) and a decrease in the frequency of hospitalizations for the entire sample (p=0.006). There was a reduction in the frequency of consultations at the Emergency Room among frail older adults (p=0.011).

**Conclusions:**

there was a reduction in hospitalization costs and frequency of visits to the Emergency Room after supplementary PHC.

## RESUMO

**Objetivos:**

identificar se a implementação de um sistema de Atenção Primária à Saúde (APS) suplementar possibilita a redução dos custos assistenciais de idosos com cardiopatias.

**Métodos:**

coorte retrospectiva de 223 pacientes cardiopatas com idade ≥ 60 anos. Os dados foram obtidos em registros médicos e banco de dados de custos, avaliados por período de um ano antes e após a implementação da APS. Os resultados foram expressos como media das frequências absolutas para número de internações e como gasto médio anual expressos em dólares (US$) em relação aos dados de custos.

**Resultados:**

houve redução nas despesas de internação após a implementação da APS suplementar (p=0,01) e diminuição da frequência de internação para toda a amostra (p=0,006). Houve redução na frequência de consultas no Pronto Atendimento entre os idosos frágeis (p=0,011).

**Conclusões:**

houve redução nos custos de internação e frequência de consulta no Pronto Atendimento após a APS suplementar.

## RESUMEN

**Objetivos:**

identificar si la implementación de un sistema complementario de Atención Primaria de Salud (APS) permite reducir los costos de atención a los ancianos con enfermedades del corazón.

**Métodos:**

cohorte retrospectiva de 223 pacientes con cardiopatía de edad ≥ 60 años. Los datos fueron obtenidos de prontuarios y bases de datos de costos, evaluados por un período de un año antes y después de la implementación de la APS. Los resultados se expresaron como frecuencias absolutas medias por número de hospitalizaciones y como gasto anual medio expresado en dólares (US$) en relación con los datos de costes.

**Resultados:**

hubo reducción de los gastos de hospitalización después de la implementación de la APS complementaria (p=0,01) y disminución de la frecuencia de hospitalizaciones para toda la muestra (p=0,006). Hubo reducción en la frecuencia de consultas en Emergencia entre los ancianos frágiles (p=0,011).

**Conclusiones:**

hubo una reducción en los costos de hospitalización y frecuencia de visitas a la Sala de Emergencia después de la APS complementaria.

## INTRODUCTION

The Brazilian population is aging rapidly. In 2016, life expectancy was 76.6 years, which meant an increase of 31.1 years, for both sexes, compared to 1940^(1)^. According to the World Health Organization (WHO), between 1950 and 2025, the group of older adults in the country should increase fifteen times, while the population will increase about five times^(2)^. Population aging, in particular, has been changing the epidemiological profile in Brazil, with an increase in mortality due to chronic noncommunicable diseases (NCDs), to the detriment of infectious and parasitic diseases^(3)^.

Among NCDs, cardiovascular diseases have the highest morbidity and mortality rates in Brazil and worldwide^(4)^. Among the main chronic cardiovascular diseases are coronary artery disease (CAD) and heart failure (HF). These diseases, in addition to being highly disabling, lead the demand for medical services, especially in older adults^(4-5)^. In 2015, specifically, CAD occupied a prominent position and was responsible for 47.7% of global cardiovascular mortality^(6)^.

Brazil has a health care model in which public and private investments coexist in provision, demanding the use of health services. The private segment is called supplementary health and is made up of health insurance operators, which, through the payment of private insurance, contribute to minimizing the public system overload^(7)^. The public system is represented by the Unified Health System (SUS - *Sistema Único de Saúde*), open for access by all Brazilians to health services. Both the public system and the supplementary health system can offer a specific model of organization of care and health services, called Primary Health Care (PHC)^(7)^. PHC has four essential attributes: first contact access, coordination, longitudinality and comprehensiveness. It is responsible for acting as a gateway to the health system, facilitating the way for patients to find the right professional at the right time and in the right place.

The São Francisco Xavier Foundation (FSFX), in December 2014, started its operations in the first PHC Unit called *Usifamília*, in the city of Ipatinga-MG, Brazil, inspired by the Cambridge Health Alliance Primary Care model, Boston, United States of America^(8)^. This resulted in a mixed model of health care based on PHC assumptions and the paradigm of older adults’ health, called health management centered on older adults^(8)^. This model aims to better manage the health of beneficiaries and their families, through a technically qualified team and the PHC model, a differentiated physical structure and a patient-centered care flow^(8)^. The health team that makes up this supplementary PHC is made up of doctors, nurses and nursing technicians, and the multidisciplinary team is made up of a nutritionist, pharmacist, social and administrative worker. *Usifamília* units have a high standard service structure with individualized service and modern equipment that meet the needs of its users. Professionals use the electronic medical record, the same used by the hospital in the FSFX network, facilitating the referral and counter-referral with the health team in information processing. Patients are actively monitored and systematically followed up. Strategies that strengthen PHC capacity and emphasize health promotion tend to improve health status and reduce costs with hospitalizations and medical procedures^(9)^. However, in order to have a broad view of the benefits generated by the implementation of supplementary PHC programs, it is still necessary to develop methods for assessing and quantifying the results obtained to justify the investment in this care model.

## OBJECTIVES

To identify whether implementing a supplementary PHC system makes it possible to reduce care costs for older adults with heart diseases.

## METHODS

### Ethical aspects

The study was conducted in accordance with national and international ethical guidelines, being approved by the Research Ethics Committee of the *Faculdade de Ciências Médicas de Minas Gerais* (FCM-MG), in November 2016.

### Study design, period, and place

This is a retrospective cohort that included all subjects aged ≥ 60 years previously diagnosed with heart failure (HF), unstable angina or who had already had an acute myocardial infarction (AMI) and who had their first medical consultation at supplementary PHC *Usifamília* from 01/01/2015 to 12/31/2015. Also considering that the classification of functionality of older adults is relevant, as it can provide support to guide quality care in health services, in this work we used the Clinical-Functional Vulnerability Index-20 (IVCF-20 - *Índice de Vulnerabilidade Clínico-Funcional-20*) to classify older adults according to their functional capabilities^(10)^. All costs with medical care and procedures were surveyed one year before and one year after the first consultation at PHC. Data collection was obtained in 2017. To prepare the manuscript, the STrengthening the Reporting of OBservational studies in Epidemiology (STROBE) recommendations were followed, being carried out based on patients’ electronic medical record and the total care cost (hospital, outpatient and home), described in the Electronic Data Interchange (EDI)^(11)^.

### Sample, and inclusion and exclusion criteria

Electronic medical records of patients who did not have heart disease described at least one year before the first medical consultation at PHC as well as those who did not have at least 12 months of follow-up before and after the first medical consultation at PHC and those with incomplete record. According to the exclusion criteria, 223 medical records of patients were selected.

### Study protocol

Data were obtained from the electronic medical record, which analyzes the medical evolution of the first consultation at PHC, and from consultations with a cardiologist before that date. Selected variables were sex, age, weight, height, smoking, clinical history (HF, angina, AMI), date of first consultation at PHC, vulnerability index according to IVCF-20, total cost of care (hospital, outpatient and home) and frequency of use of Emergency Room and hospital admissions.

In Brazil, PHC professionals tend to consider older adults as fragile based on their general appearance, or when these individuals have multiple illnesses or comorbidities. The IVCF-20 is an index of vulnerability of older adults obtained through a questionnaire that considers multidimensional aspects of the health condition, being considered a screening and classification tool for frailty and loss of functional capacity in older adults^(10)^. The IVCF-20 is useful to be used for initial screening in PHC and represents a systematic attempt to objectively verify the ability of an individual to perform the activities necessary for self-care, based on the assessment of different skills, allowing the development of an interdisciplinary health care plan especially aimed at optimizing older adults’ performance^(10)^.

The questionnaire consists of twenty questions, distributed in different domains: age, self-perceived health, functional impairment, cognition, mood, mobility, communication and multiple comorbidities^(10)^. Each domain has a specific score that makes up a maximum value of forty points. The higher the value obtained, the greater the risk of clinical and functional vulnerability of older adults, which can be classified: 0 to 6 points: (robust older adults) they can exercise their autonomy and independence and develop all their activities of daily living independently, without showing functional disability; 7 to 14 points: (older adults at risk of frailty) present a decrease in their functional clinical condition, i.e., physical and mental weakness, but present themselves as independent and autonomous; and ≥ 15 points: (frail older adults) semi-dependent or totally dependent on their activities, i.e., they are unable to manage their lives independently and autonomously^(10)^. At PHC *Usifamília*, this questionnaire is routinely applied by duly trained nursing technicians and later checked by the unit’s nurse. Application time is approximately 10 minutes.

To calculate the BMI, the cut-off points proposed by Lipschitz and the Ministry of Health were used, taking into account the changes in body composition that occur with aging, when compared with adult individuals, according to the cut-off points proposed by the WHO^(12-13)^. Individuals were classified as underweight, with a BMI ≤ 22 kg/m^2^, eutrophic or adequate weight, with a BMI between 22 kg/m^2^ and 27 kg/m^2^, and overweight, with a BMI ≥ 27 kg/m^2(12-13)^.

For cost assessment (hospital, outpatient and home), the average annual costs in the 12-month periods prior to the date of the first appointment at PHC and 12 months after were considered (we remember that, in the following 12 months, participants continued to be followed up by supplemental PHC). Outpatient costs refer to elective medical consultations, Emergency Room consultations and laboratory tests. There was no monetary correction of values, since each patient in the sample had a different admission date at PHC. Only direct costs were taken into account in this study. All costs were originally calculated in the national currency (reais). These amounts were then converted into US dollars, according to data provided by the Central Bank corresponding to 10/04/2020 (1US$ =4.60 BRL).

### Analysis of results, and statistics

Qualitative variables were presented as absolute and relative frequencies, and quantitative variables as mean ± standard deviation and median. Quantitative variables were submitted to the Shapiro-Wilk normality test. To assess the association between categorical variables, the chi-square test of independence was used. Comparison of three or more means was performed using the Kruskal-Wallis test, and for multiple comparisons, the Dunn test was adopted. Comparison of expenses before and after PHC implementation was performed using the Wilcoxon test for paired samples. Analyzes were developed in the free program R version 3.2.2, adopting a significance level of 5%.

## RESULTS

The sample consisted of 223 participants, of which 60.1% were male. The mean age was 73.84 ± 7.89 years, of which 40.4% were between 60 and 70 years old. The mean BMI was 29.14 ± 4.87 kg/m^2^, and 67.5% were overweight. Regarding clinical history, 77.1% had AMI, 18.4%, HF, and 9%, angina. Only 9.6% were smokers (Table 1).

**Table 1:** Characteristics of older adult cardiac patients attended at the primary health service, Ipatinga, Minas Gerais, Brazil, 2015

Characteristics	Entire sample (Nº 223)	IVCF-20 classification			*p* value
		Frail (Nº 25)	At risk (N^o^ 85)	Robust (Nº 113)	
					
Sex					<**0,001** ^Q^
Female	89(39.9%)	15(60%)	48(56.5%)	26(23%)	
Male	134(60.1%)	10(40%)	37(43.5%)	87(77%)	
					
Age (years)	73.84 ± 7.89	82.16 ± 7.40^†.€^	74.85 ± 7.49^†.₵^	71.24 ± 6.82^€.₵^	<**0.001** ^K^
60 to 70 years	90(40.4%)	1(4%)	34(40%)	55(48.7%)	
71 to 80 years	83(37.2%)	11(44%)	24(28.2%)	48(42.5%)	
81 years and older	50(22.4%)	13(52%)	27(31.8%)	10(8.8%)	
					
^BMI (kg/m2)#^	29.14 ± 4.87	30.83 ± 5.26	29.40 ± 5.11	28.61 ± 4.54	0.110^K^
Underweight	11(5.3%)	-	7(9%)	4(3.8%)	
Eutrophic	56(27.2%)	5(22.7%)	15(19.2%)	36(34%)	
Overweight	139(67.5%)	17(77.3%)	56(71.8%)	66(62.3%)	
					
Clinical history					
HF	41(18.4%)	11(44%)	14(16.5%)	16(14.2%)	0.002^Q^
Angina	20(9%)	4(16%)	8(9.4%)	8(7.1%)	0.363^Q^
AMI	172(77.1%)	12(48%)	65(76.5%)	95(84.1%)	<**0.001** ^Q^
					
Smoker	18(9.6%)	1(4.8%)	7(10%)	10(10.4%)	<**0.001** ^Q^
					

IVCF-20 - Clinical-Functional Vulnerability Index-20; HF - heart failure; AMI - acute myocardial infarction; BMI - Body Mass Index. #Variable has missing data (BMI n=17, smoking n=36). P-values refer to the

Q Chi-square test of independence or

K Kruskal-Wallis, with multiple Dunn comparisons. The symbols

†,€, ^₵^ indicate pairs with significant differences.

Regarding the IVCF-20 classification, 25 older adults were considered frail, 85 at risk of frailty, and 113 robust. Frail older adults were older (p<0.001), with a higher proportion of women (p<0.001), with HF (p=0.002) and a lower proportion of AMI (p<0.001). Robust older adults had a higher proportion of men (p<0.001), with AMI (p<0.001) and smokers (p <0.001) (Table 1).

The reduction in the cost of total health care after implementing PHC was not significant for the entire sample (mean ± SD, US$ 269.90 ± US$ 2,517.43, p=0.368) nor among the analyzed subgroups. There was no significant difference in total cost reduction by sex, age, medical history, BMI, smoking, and IVCF-20 classification (Table 2).

**Table 2:** Average annual costs for health care and expenses with hospitalizations before and after implementing the Primary Health Care service according to the characteristics of older adult patients with heart disease, Ipatinga, Minas Gerais, Brazil, 2015

Characteristics	Average annual cost of spending on health care			Average annual cost of spending on admissions		
	Before (US$)	After (US$)	*p* value	Before (US$)	After (US$)	*p* value
Entire sample	1,116.79 ± 2,025.22(276.74)	846.88 ± 1,644,77(245.05)	0.368^Wp^	1,936.14 ± 2,520.72(972.98)	1,237.71 ± 2,130.92(303.43)	0.011^Wp^
Sex						
Female	1,327.81 ± 2,215.74(421.93)	1,041,81 ± 1,899.00(284.99)	0.515^Wp^	2,024.55 ± 2,579.98(774.49)	1,377.02 ± 2,278.90(664.58)	0.150^Wp^
Male	976.63 ± 1,883.64(234.77)	717.41 ± 1,444.71(192.81)	0.574^Wp^	1,860.58 ± 2,490.30(1,166,70)	1,118.67± 2,009.41(0)	0.041^Wp^
Age (years)						
60 to 70 years	1,032.17 ± 1,933.93(256.93)	701.00 ± 1,461.62(194.65)	0.652^Wp^	1,920.86 ± 2,527.21(552.80)	1,053.78 ± 2,060.63(0)	0.129^Wp^
71 to 80 years	1,150.04 ± 1,971.11(395.38)	664.73 ± 1,170.18(219.61)	0.189^Wp^	1,789.95 ± 2,451.27(1,175.35)	705.22 ± 1,548.91(0)	0.005^Wp^
81 years and older	1,213.89 ± 2,294.02(231.96)	1411.83 ± 2,379.85(286.58)	0.623^Wp^	2,209.42± 2,709.13(821.80)	2,430.94 ± 2,660.08(1,515.57)	0.944^Wp^
Clinical history						
HF	1,648.11 ± 2,801.57(479.76)	1,324.32 ± 2,161,07(477.07)	0.564^Wp^	2,275.03 ± 3,244.79(935.74)	1,612.62 ± 2,285.84(749.01)	0.328^Wp^
Angina	1,582.21 ± 2,319.09(597.65)	1,087.46 ± 2,208,26(355.95)	0.189^Wp^	2,410.56 ± 2,694.63(1,182.49)	1,514.63 ± 2,870.70(0)	0.320^Wp^
AMI	943.19 ± 1,703.63(232.88)	693.29 ± 1,350.04(204.52)	0.766^Wp^	1,769.19 ± 2,124.50(954.58)	1,048.42 ± 1,877.60(0)	0.023^Wp^
BMI						
Underweight	613.27 ± 122,58(200.72)	567.50 ± 735.81(189.85)	0.465^Wp^	1,573.91 ± 2,270.97(544.45)	761.39 ± 725.57(839.32)	0.750^Wp^
Eutrophic	882.18 ± 1,217.05(365.23)	612.09 ± 1,124.86(220.18)	0.107^Wp^	1,451.90± 1,415.93(1,219.43)	792.96 ± 1,454.39(0)	0.027^Wp^
Overweight	1,198.25 ± 2,248.55(258.50)	958.90 ± 1,872.09(254.15)	0.935^Wp^	2,005.38± 12.779.82(869.10)	1,386.25 ± 2,392.71(281.93)	0.217^Wp^
Smoker						
No	1,121.19 ± 2,089.31(279.31)	798.86 ± 1,535.68(257.11)	0.362^Wp^	1,997.85 ± 2,652.08(972.98)	1,184.11 ± 2,115.79(401.92)	0.020^Wp^
Yes	828.03 ± 1,692.24(204.00)	685.56 ± 1,272.09(93.35)	0.393^Wp^	2,015.16± 2,505.79(855.58)	1,173.43 ± 1,590.93(318.44)	0.563^Wp^
IVCF-20 classification						
Frail	2,691.82 ± 3,451.45(1,214.08)	1,813,05 ± 2,726.62(689.75)	0.300^Wp^	3,333.12 ± 3,595.54(2,338.10)	2,133.13 ± 2,958.77(835.27)	0.275^Wp^
At risk	894.95 ± 1,747.32(253.00)	856.45 ± 1,663.13(245.06)	0.671^Wp^	1,391.63 ± 2,117.40(529.47)	1,185.36 ± 2,010.89(527.12)	0.556^Wp^
Robust	935,19 ± 1,631.83(236.93)	625.92 ± 1,198.72(196.37)	0.340^Wp^	1,806.11± 2,100.12(1,182.49)	903.85 ± 1,725.99(0)	0.013^Wp^

IVCF-20 - Clinical-Functional Vulnerability Index-20; HF - heart failure; AMI - acute myocardial infarction; BMI - Body Mass Index. Data are presented as mean ± SD (median). #Difference= cost before - cost after;

W Wilcoxon Mann-Whitney test for independent samples;

Wp Wilcoxon test for paired samples;

K Kruskal-Wallis test.

However, there was a significant reduction in hospitalization costs after implementing a PHC for the entire sample (mean ± SD, US$ 698.43 ± US$ 3,500.76, p=0.011). When stratifying the groups, there was a significant difference in hospitalization costs for men (p=0.041), between 71 and 80 years old (p=0.005), who had AMI (p=0.023), eutrophic according to BMI (p= 0.027), non-smokers (p=0.020) and classified as robust according to IVCF-20 (p=0.013) (Table 2).

There was a reduction in the frequency of hospital admissions with PHC implementation for the entire sample (p=0.006), and among men (p=0.014), between 71 and 80 years old (p=0.001), classified as robust by IVCF-20 (p=0.025) and who had already been diagnosed with AMI (p=0.027). There was a reduction in the frequency of consultations in the Emergency Room among older adults classified as frail in IVCF-20 (p=0.011) (Table 3).

**Table 3:** Annual frequency of hospital admissions and Emergency Room consultations, before and after implementing a Primary Health Care according to sex, age, Clinical-Functional Vulnerability Index-20 rate and clinical history, Ipatinga, Minas Gerais, Brazil, 2015

Characteristics	N^o^ of admissions			N^o^ of Emergency Room consultations		
	Before	After	*p* value	Before	After	*p* value
Entire sample	1.25 ± 1.31(1)	0.77 ± 0.90(1)	0.006	1.36 ± 1.42(1)	1.06 ± 1.09(1)	0.139
Sex						
Female	1.28 ± 1.21(1)	0.96 ± 1.02(1)	0.165	1.55 ± 1.43(1)	1.16 ± 1.15(1)	0.162
Male	1.22 ± 1.40(1)	0.62 ± 0.76(0)	0.014	1.21 ± 1.40(1)	0.99 ± 1.05(1)	0.461
Age (years)						
60 to 70 years	0.84 ± 0.73(1)	0.57 ± 0.69(0)	0.180	1.09 ± 1.14(1)	1.09 ± 1.03(1)	0.919
71 to 80 years	1.61 ± 1.69(1)	0.66 ± 0.91(0)	**0.001**	1.64 ± 1.62(1)	1.02 ± 1.14(1)	0.060
81 years and older	1.25 ± 1.11(1)	1.29 ± 1.00(1)	0.774	1.23 ± 1.33 (1)	1.10 ± 1.14(1)	0.887
IVCF-20 classification						
Frail	2.32 ± 2.00(2)	1.16 ± 1.07 1)	0.086	2.76 ± 2.08(2)	0.88 ± 1.32(0)	0.011
At risk	0.95 ± 0.80(1)	0.79 ± 0.78(1)	0.430	1.16 ± 1.08(1)	1.06 ± 1.01(1)	0.581
Robust	1.04 ± 1.07(1)	0.60 ± 0.89(0)	0.025	1.14 ± 1.24(1)	1.11 ± 1.11(1)	1.000
Clinical history						
HF	1.77 ± 2.12(1)	1.23 ± 1.14(1)	0.472	1.78 ± 1.98(1)	1.19 ± 1.35(1)	0.267
Angina	1.45 ± 0.69(2)	0.91 ± 1.14(0)	0.224	1.73 ± 1.33(1)	1.33 ± 1.18(1)	0.492
AMI	1.07 ± 1.00(1)	0.67 ± 0.78(0)	0.027	1.21 ± 1.23(1)	0.98 ± 0.97(1)	0.286

*IVCF-20 - Clinical-Functional Vulnerability Index-20; HF- heart failure; AMI - acute myocardial infarction; BMI - Body Mass Index. Data are presented as mean ± SD (median). P-values refer to the Wilcoxon Test for paired samples.*

## DISCUSSION

In this study, patients were mostly male, and the mean age was 73.8, of which (40.4%) were between 60 and 70 years old. The majority (67.5%) were overweight. More patients with AMI were observed (77.1%), and most were classified as robust according to the IVCF-20 (50.7%). The frail older adults were older and mostly female (60%), with HF (lesser proportion with AMI). After implementing a PHC for frail older adults, there was a reduction in the number of medical consultations in Emergency Room Units, and for robust older adults, a reduction in the number of hospitalizations. Although the reduction in total cost after implementing the supplementary PHC was not significant, a significant reduction in costs and frequency of hospital admissions was observed for the entire sample. When stratifying the groups, there was a significant difference in hospitalization costs for men, aged between 71 and 80 years, who had AMI, eutrophic according to BMI, non-smokers and classified as robust according to IVCF-20. Regarding the frequency of hospitalization, there was a reduction with the implementation of a PHC for the entire sample and among men, aged between 71 and 80 years, classified as robust by the IVCF-20 and diagnosed with AMI. A reduction in the frequency of Emergency Room consultations was also observed among older adults classified as frail in IVCF-20. The results show the importance of implementing the supplementary PHC, both in terms of health and the system’s financial sustainability, by avoiding hospitalizations and also possible health complications in a population of older adults with heart disease who are more vulnerable.

As for the results of this study, and for comparison purposes, it is interesting to mention that the reduction in hospitalizations was also observed by another supplementary health care provider in the city of Belo Horizonte, Minas Gerais, Brazil, which recorded a 13% reduction in hospitalizations after implementing PHC^(14)^. However, it is noteworthy that, in this case, contrary to the practice of this study, the results were obtained among all individuals, without any type of classification, and which constituted a certain portfolio of clients in relation to all individuals in the portfolio of another client without the implementation of a PHC, not following the same individual, as in the case of this study. However, both in the case of PHC *Usifamília* and that of the referred operator, we observed the impact of PHC in the reduction of hospital admissions.

We know that many chronic conditions are linked to aging, but also derive from the lifestyle of citizens, characterized by unhealthy habits, in addition to genetic predisposition^(3)^. A higher proportion of overweight older adults (67.5%) was observed in this study, when compared to data obtained from a survey carried out by the Ministry of Health, published in 2020, in the bulletin Vigitel (*Vigilância de fatores de risco e proteção para doenças crônicas por inquérito telefônico*), in which 60.9% of older adults aged 65 years or older were overweight^(15)^. Regarding smoking, the proportion was lower (9.6%), compared to the 2013 data from the Brazilian Institute of Geography and Statistics (IBGE - *Instituto Brasileiro de Geografia e Estatística*) (13.7%) for the Southeast^(16)^. It is known that the control of risk factors is responsible for at least 50% of reduction in mortality from cardiovascular diseases^(17)^.

Like AMI, unstable angina and HF are the most prevalent NCDs in older adults and with high morbidity and mortality^(18-26)^. AMI had the highest proportion between the groups (77.1%), followed by HF (18.4%) and angina (9%). HF showed a higher proportion in frail older adults, an expected fact, since HF is a predictor of dependence in hospitalized older adults and increases the chance of functional loss fivefold^(6)^. In our study, the costs of caring for patients with HF were higher than the costs of treating conditions related to AMI or angina. This was already expected, since HF expenses tend to be higher, because they entail a series of consequences, such as pulmonary edema, low perfusion of noble organs with consequent functional alterations.

There is great concern about maintaining the quality of health care for older adults without losing control of costs or failing to provide the necessary treatments. The high rates of hospitalization of older adults show the impact of the Brazilian population aging on the health sector and represent a major challenge for health systems, due to the risk of not offering the necessary assistance to the demands of this group^(3)^.

The higher health care costs for older adults in Brazil are mainly due to repeated hospitalizations^(27)^. In 2009, older adults accounted for 21% of hospitalizations^(28)^. The main causes of hospitalizations for PHC-sensitive conditions reported for older adults are HF, angina, pulmonary diseases and cerebrovascular diseases^(29)^.

Analyzing data on hospitalizations of older adults in 2014, in Brazilian private hospitals that serve the supplementary health network, it is observed that the main causes of hospitalization among older adults were diseases of the circulatory system^(3)^. In a study carried out with older adults of PHC in the SUS, diseases of the circulatory system were also the main cause of hospitalization (28.4%)^(28)^. Hospitalizations for HF and myocardial revascularization surgeries were responsible for the largest share of SUS costs^(24)^. From 1998 to 2010, DATASUS (SUS IT department) estimated a historical series of hospitalizations due to AMI, angioplasty and revascularization, bringing an average cost per patient to US$ 1,138.26 in the public system and US$ 3,675.00 in the systems private^(28)^. It is noteworthy that the average cost of hospitalization due to AMI before implementing PHC *Usifamília* in our study was US$ 1,769.18 ± 2,124.49 and represents an intermediate value between those mentioned in the analysis, being above for the SUS and below for private systems. On the other hand, the average cost of US$ 1,048.42 ± 1,877.60 (p=0.023), with the same type of hospitalization, after implementing PHC *Usifamília*, shows a clear reduction in relation to those mentioned in the SUS and in private systems.

In this context, monitoring older adults in PHC services proposes patient-focused care, in order to reduce the frequency of hospitalizations and/or reduce the demand for Emergency Room services. Avoiding hospitalizations in older adults is relevant both for health and quality of life issues and for cost reduction.

We observed a significant reduction in hospitalization costs after implementing a PHC for the entire sample and in men, aged between 71 and 80 years, with a previous diagnosis of AMI, eutrophic BMI, non-smokers and classified as robust. Moreover, after implementing PHC, it was observed that there was no increase in total costs, but a significant reduction in the need for hospitalization and, consequently, a reduction in hospitalization costs (it is worth remembering that the total costs after the implementation of this work include PHC service costs). Implementing PHC *Usifamília* allowed for greater investment in elective consultations, diagnostic tests, prevention, reflecting on better health. These actions, taken together, make it possible to improve the health system efficiency and allow for an improvement in older adults’ quality of life.

It was also verified, in this study, that frail older adults had a reduction in the number of medical consultations in Emergency Room Units (p=0.001) and lower costs (total and with hospitalizations) with PHC implementation. However, no statistically significant differences were observed in these last variables for the group of individuals. It can be inferred, from the results obtained, that frail older adults are more subject to prolonged hospitalizations, successive readmissions and worse prognosis.

This study shows the importance of implementing a PHC network to care for older adults with heart disease, since not only do we have a reduction in health costs, but also a decrease in the number of hospitalizations and emergency room visits. These are data that not only help in the strategic economic planning for implementing health programs, but also allow inferring an improvement in the quality of life of this population.

### Study limitations

Only older adults with a diagnosis registered in the medical records of AMI, HF and angina in the evolution of the first PHC consultation were included in the study. It is possible to observe that some older adults who had these heart diseases were excluded from the study due to lack of records of the evolution at the time of the first consultation. Some subjects probably developed HF secondary to an AMI. However, we did not verify AMI history in these cases, and the starting point for their allocation in the group was the diagnosis declared in this 1^st^ consultation at PHC.

### Contributions to nursing and health

The results allow us to broaden our understanding of the care network for older adults, enabling planning strategies to identify older adults with the potential for hospitalization and Emergency Room consultations.

## CONCLUSIONS

A clear reduction in the frequency and costs of hospitalization was observed for older adult patients with heart disease who were previously treated in supplementary PHC. Although the reduction in the total health cost was not statistically significant for the entire sample, we must remember that costs with services provided by supplementary PHC were taken into account, showing that expenses with the service implementation did not burden the health system. Our results as a whole suggest that implementing the PHC improved quality of care for older adults diagnosed with heart disease. It is evident, therefore, the need to act to focus PHC teams on monitoring older adults, especially with regard to monitoring the time of return to care, in addition to intensifying preventive actions in order to identify early risk factors for cardiovascular diseases.

## Supplementary Material

0034-7167-reben-76-03-e20220486-sup01

## Data Availability

https://doi.org/10.48331/scielodata.XY1TB4.
