# Supplementary material for: Primary care in supplementary health: assessment of costs in the care of older adult patients with heart diseases
Source: Rev Bras Enferm. 2023 Jul 10;76(3):e20220486. doi: 10.1590/0034-7167-2022-0486 (PMC10332371; doi:10.1590/0034-7167-2022-0486)
Supplement: 0034-7167-reben-76-03-e20220486-sup01 [file 0034-7167-reben-76-03-e20220486-sup01.pdf]

IDENTIFICACAO DO PARTICIPANTE	SEXO	DT_NASCIMENTO	IDADE	STATUS_PLANO	STATUS_BENEFICIARIO	DT_1ºATENDIMENTO
47863	FEMININO	2/17/1935	82	INATIVO	OBITO	8/31/2015
42781	MASCULINO	4/12/1952	65	ATIVO	PARTICIPANTE	5/14/2015
91497	FEMININO	9/27/1929	87	INATIVO	OBITO	7/7/2015
44018	MASCULINO	4/25/1935	82	INATIVO	EXCLUIDO DO PLANO	4/16/2015
57027	FEMININO	12/26/1951	65	ATIVO	PARTICIPANTE	3/19/2015
64073	MASCULINO	7/4/1934	82	ATIVO	PARTICIPANTE	3/16/2015
60430	MASCULINO	12/26/1952	64	ATIVO	PARTICIPANTE	11/5/2015
42548	FEMININO	5/17/1931	86	ATIVO	PARTICIPANTE	1/5/2015
93948	MASCULINO	4/7/1936	81	ATIVO	PARTICIPANTE	10/27/2015
48157	MASCULINO	8/2/1950	66	ATIVO	PARTICIPANTE	12/28/2015
45648	FEMININO	3/28/1941	76	ATIVO	PARTICIPANTE	8/5/2015
44606	MASCULINO	8/19/1932	84	ATIVO	PARTICIPANTE	2/24/2015
43436	MASCULINO	11/14/1940	76	ATIVO	PARTICIPANTE	9/28/2015
48668	FEMININO	11/26/1948	68	ATIVO	PARTICIPANTE	9/23/2015
45620	MASCULINO	12/20/1938	78	ATIVO	PARTICIPANTE	1/6/2015
69299	FEMININO	7/7/1938	78	INATIVO	OBITO	1/21/2015
489208	FEMININO	8/1/1934	82	ATIVO	PARTICIPANTE	9/29/2015
94798	MASCULINO	6/21/1938	78	ATIVO	PARTICIPANTE	7/1/2015
44098	MASCULINO	3/31/1942	75	ATIVO	PARTICIPANTE	3/12/2015
56905	MASCULINO	12/17/1955	61	ATIVO	PARTICIPANTE	6/2/2015
65847	FEMININO	12/8/1944	72	ATIVO	PARTICIPANTE	1/20/2015
47610	MASCULINO	1/3/1946	71	ATIVO	PARTICIPANTE	3/3/2015
44256	MASCULINO	9/11/1950	66	INATIVO	OBITO	3/19/2015
43533	FEMININO	6/12/1934	82	ATIVO	PARTICIPANTE	2/4/2015
49713	FEMININO	3/19/1947	70	ATIVO	PARTICIPANTE	2/26/2015
47341	FEMININO	10/30/1951	65	ATIVO	PARTICIPANTE	3/9/2015
57900	FEMININO	2/10/1935	82	ATIVO	PARTICIPANTE	6/1/2015
42663	MASCULINO	8/17/1951	65	ATIVO	PARTICIPANTE	10/3/2015
49002	FEMININO	2/6/1939	78	ATIVO	PARTICIPANTE	2/19/2015
53974	MASCULINO	4/26/1927	90	ATIVO	PARTICIPANTE	9/22/2015
48781	FEMININO	2/8/1948	69	ATIVO	PARTICIPANTE	10/8/2015
63400	FEMININO	8/15/1942	74	ATIVO	PARTICIPANTE	12/28/2015
47752	MASCULINO	12/29/1940	76	ATIVO	PARTICIPANTE	8/10/2015
84272	MASCULINO	2/14/1933	84	ATIVO	PARTICIPANTE	1/13/2015
44224	MASCULINO	5/17/1948	69	ATIVO	PARTICIPANTE	8/27/2015
47817	FEMININO	4/14/1943	74	ATIVO	PARTICIPANTE	2/20/2015
45116	FEMININO	2/5/1947	70	ATIVO	PARTICIPANTE	7/17/2015
88815	FEMININO	2/25/1952	65	ATIVO	PARTICIPANTE	6/29/2015
63417	MASCULINO	4/26/1948	69	INATIVO	OBITO	5/29/2015
51330	FEMININO	9/16/1942	74	ATIVO	PARTICIPANTE	9/1/2015
46173	MASCULINO	7/29/1939	77	ATIVO	PARTICIPANTE	2/2/2015
57614	MASCULINO	12/3/1944	72	INATIVO	OBITO	2/27/2015
43572	FEMININO	9/6/1944	72	ATIVO	PARTICIPANTE	2/4/2015
50168	FEMININO	7/7/1939	77	ATIVO	PARTICIPANTE	8/31/2015
1072442	MASCULINO	10/12/1955	61	ATIVO	PARTICIPANTE	12/16/2015
44831	MASCULINO	2/10/1944	73	INATIVO	EXCLUIDO DO PLANO	9/10/2015
46897	MASCULINO	2/5/1944	73	ATIVO	PARTICIPANTE	9/17/2015
47087	FEMININO	2/15/1950	67	ATIVO	PARTICIPANTE	10/16/2015
58335	MASCULINO	10/25/1949	67	ATIVO	PARTICIPANTE	11/6/2015
56030	MASCULINO	8/14/1949	67	ATIVO	PARTICIPANTE	5/4/2015
99173	FEMININO	11/11/1934	82	ATIVO	PARTICIPANTE	1/7/2015
94714	FEMININO	4/30/1947	70	ATIVO	PARTICIPANTE	11/17/2015
48420	MASCULINO	3/18/1942	75	ATIVO	PARTICIPANTE	2/10/2015

51903	MASCULINO	11/13/1945	71	ATIVO	PARTICIPANTE	7/10/2015
50862	MASCULINO	4/19/1949	68	ATIVO	PARTICIPANTE	5/19/2015
59648	FEMININO	7/12/1949	67	ATIVO	PARTICIPANTE	3/11/2015
82910	MASCULINO	2/27/1949	68	ATIVO	PARTICIPANTE	6/1/2015
48391	FEMININO	11/22/1947	69	ATIVO	PARTICIPANTE	9/17/2015
65183	MASCULINO	7/12/1952	64	ATIVO	PARTICIPANTE	8/17/2015
45663	MASCULINO	5/9/1934	83	ATIVO	PARTICIPANTE	1/30/2015
47649	MASCULINO	6/7/1938	78	ATIVO	PARTICIPANTE	7/22/2015
48646	MASCULINO	4/29/1946	71	ATIVO	PARTICIPANTE	9/29/2015
44715	FEMININO	12/5/1935	81	ATIVO	PARTICIPANTE	8/6/2015
48490	MASCULINO	8/11/1938	78	ATIVO	PARTICIPANTE	4/9/2015
46238	MASCULINO	5/24/1933	84	ATIVO	PARTICIPANTE	5/8/2015
45020	MASCULINO	3/24/1947	70	ATIVO	PARTICIPANTE	3/27/2015
56637	FEMININO	1/5/1957	60	ATIVO	PARTICIPANTE	6/15/2015
96231	MASCULINO	4/12/1944	73	ATIVO	PARTICIPANTE	3/30/2015
49088	MASCULINO	11/19/1944	72	ATIVO	PARTICIPANTE	3/4/2015
66974	FEMININO	6/28/1954	62	ATIVO	PARTICIPANTE	5/14/2015
44048	MASCULINO	4/4/1936	81	ATIVO	PARTICIPANTE	10/20/2015
48140	MASCULINO	5/2/1939	78	ATIVO	PARTICIPANTE	8/20/2015
52161	FEMININO	1/17/1934	83	ATIVO	PARTICIPANTE	10/1/2015
43745	MASCULINO	1/10/1942	75	ATIVO	PARTICIPANTE	9/28/2015
51133	FEMININO	4/11/1940	77	ATIVO	PARTICIPANTE	3/24/2015
51271	FEMININO	7/28/1938	78	ATIVO	PARTICIPANTE	7/29/2015
48819	FEMININO	11/14/1947	69	ATIVO	PARTICIPANTE	5/7/2015
50605	MASCULINO	11/28/1940	76	ATIVO	PARTICIPANTE	3/11/2015
54694	MASCULINO	6/7/1950	66	ATIVO	PARTICIPANTE	12/31/2015
51923	FEMININO	2/5/1954	63	ATIVO	PARTICIPANTE	5/26/2015
46085	MASCULINO	4/16/1948	69	INATIVO	EXCLUIDO DO PLANO	6/29/2015
52230	MASCULINO	4/15/1956	61	ATIVO	PARTICIPANTE	10/21/2015
49231	MASCULINO	9/13/1950	66	ATIVO	PARTICIPANTE	11/25/2015
45316	FEMININO	1/1/1946	71	ATIVO	PARTICIPANTE	1/26/2015
48497	FEMININO	3/12/1928	89	ATIVO	PARTICIPANTE	1/15/2015
114606	MASCULINO	3/23/1951	66	ATIVO	PARTICIPANTE	6/16/2015
51794	FEMININO	9/14/1953	63	ATIVO	PARTICIPANTE	5/6/2015
47445	MASCULINO	11/1/1941	75	ATIVO	PARTICIPANTE	8/31/2015
51067	MASCULINO	8/27/1944	72	ATIVO	PARTICIPANTE	2/24/2015
46084	MASCULINO	3/23/1947	70	ATIVO	PARTICIPANTE	11/27/2015
50017	MASCULINO	5/28/1939	78	ATIVO	PARTICIPANTE	9/24/2015
67832	MASCULINO	4/20/1955	62	ATIVO	PARTICIPANTE	11/23/2015
44360	MASCULINO	8/24/1946	70	ATIVO	PARTICIPANTE	3/4/2015
55459	FEMININO	5/23/1947	70	ATIVO	PARTICIPANTE	10/7/2015
43044	FEMININO	10/30/1934	82	ATIVO	PARTICIPANTE	7/31/2015
94244	MASCULINO	11/17/1941	75	ATIVO	PARTICIPANTE	9/23/2015
42961	MASCULINO	12/7/1933	83	ATIVO	PARTICIPANTE	3/4/2015
61123	MASCULINO	5/22/1929	88	ATIVO	PARTICIPANTE	12/4/2015
104078	MASCULINO	4/6/1949	68	ATIVO	PARTICIPANTE	7/9/2015
57814	MASCULINO	5/17/1951	66	ATIVO	PARTICIPANTE	7/31/2015
72497	MASCULINO	1/16/1956	61	ATIVO	PARTICIPANTE	10/6/2015
58332	MASCULINO	10/6/1951	65	ATIVO	PARTICIPANTE	6/8/2015
70742	FEMININO	5/3/1933	84	ATIVO	PARTICIPANTE	1/6/2015
62715	MASCULINO	10/23/1946	70	ATIVO	PARTICIPANTE	6/3/2015
65926	MASCULINO	7/22/1939	77	ATIVO	PARTICIPANTE	3/30/2015
42981	MASCULINO	10/16/1943	73	ATIVO	PARTICIPANTE	2/12/2015
62530	MASCULINO	12/5/1941	75	ATIVO	PARTICIPANTE	3/27/2015
108308	MASCULINO	8/20/1934	82	ATIVO	PARTICIPANTE	2/10/2015
73654	MASCULINO	8/13/1944	72	ATIVO	PARTICIPANTE	3/2/2015

107721	FEMININO	9/28/1921	95	ATIVO	PARTICIPANTE	2/9/2015
100651	MASCULINO	11/8/1932	84	ATIVO	PARTICIPANTE	8/11/2015
110437	MASCULINO	10/21/1936	80	ATIVO	PARTICIPANTE	1/16/2015
66431	FEMININO	2/28/1939	78	ATIVO	PARTICIPANTE	3/9/2015
89409	MASCULINO	8/2/1923	93	ATIVO	PARTICIPANTE	2/5/2015
96363	FEMININO	8/14/1934	82	ATIVO	PARTICIPANTE	11/17/2015
109326	FEMININO	2/10/1947	70	ATIVO	PARTICIPANTE	9/9/2015
44937	MASCULINO	5/25/1956	60	ATIVO	PARTICIPANTE	4/23/2015
73441	MASCULINO	11/14/1948	68	ATIVO	PARTICIPANTE	7/29/2015
80700	FEMININO	3/4/1936	81	ATIVO	PARTICIPANTE	11/30/2015
85754	FEMININO	5/18/1927	90	ATIVO	PARTICIPANTE	3/25/2015
103902	MASCULINO	6/13/1946	70	ATIVO	PARTICIPANTE	4/27/2015
49854	MASCULINO	7/12/1949	67	ATIVO	PARTICIPANTE	5/8/2015
43643	MASCULINO	10/15/1932	84	ATIVO	PARTICIPANTE	9/28/2015
74795	FEMININO	12/10/1950	66	ATIVO	PARTICIPANTE	6/8/2015
55660	MASCULINO	11/9/1951	65	ATIVO	PARTICIPANTE	11/3/2015
59287	MASCULINO	10/26/1955	61	ATIVO	PARTICIPANTE	12/21/2015
44821	MASCULINO	5/28/1947	69	ATIVO	PARTICIPANTE	7/1/2015
48234	MASCULINO	8/6/1956	60	ATIVO	PARTICIPANTE	12/9/2015
61091	FEMININO	8/8/1936	80	ATIVO	PARTICIPANTE	4/1/2015
50329	FEMININO	3/8/1946	71	ATIVO	PARTICIPANTE	6/13/2015
48675	FEMININO	2/11/1949	68	ATIVO	PARTICIPANTE	4/17/2015
54540	MASCULINO	6/10/1940	76	ATIVO	PARTICIPANTE	8/21/2015
46720	FEMININO	12/17/1945	71	ATIVO	PARTICIPANTE	7/21/2015
532734	MASCULINO	2/19/1931	86	ATIVO	PARTICIPANTE	1/19/2015
44341	MASCULINO	9/20/1950	66	ATIVO	PARTICIPANTE	7/6/2015
47922	MASCULINO	5/23/1934	83	ATIVO	PARTICIPANTE	1/30/2015
46592	MASCULINO	1/7/1945	72	ATIVO	PARTICIPANTE	2/12/2015
51170	FEMININO	7/4/1934	82	ATIVO	PARTICIPANTE	7/31/2015
43017	MASCULINO	5/8/1944	73	ATIVO	PARTICIPANTE	9/25/2015
45332	FEMININO	8/20/1933	83	ATIVO	PARTICIPANTE	7/24/2015
47987	MASCULINO	2/26/1936	81	ATIVO	PARTICIPANTE	2/9/2015
66154	MASCULINO	7/5/1955	61	INATIVO	EXCLUIDO DO PLANO	10/30/2015
118062	MASCULINO	6/21/1941	75	ATIVO	PARTICIPANTE	3/3/2015
46306	MASCULINO	2/19/1943	74	ATIVO	PARTICIPANTE	2/27/2015
81319	FEMININO	2/15/1947	70	ATIVO	PARTICIPANTE	5/29/2015
43630	MASCULINO	5/14/1943	74	ATIVO	PARTICIPANTE	3/19/2015
75355	MASCULINO	12/1/1954	62	ATIVO	PARTICIPANTE	10/2/2015
47977	MASCULINO	10/3/1946	70	ATIVO	PARTICIPANTE	6/26/2015
47580	MASCULINO	1/5/1937	80	ATIVO	PARTICIPANTE	7/30/2015
49078	MASCULINO	12/7/1937	79	ATIVO	PARTICIPANTE	7/29/2015
72234	MASCULINO	11/20/1956	60	ATIVO	PARTICIPANTE	11/18/2015
65689	FEMININO	9/18/1946	70	ATIVO	PARTICIPANTE	3/9/2015
48107	MASCULINO	11/4/1948	68	ATIVO	PARTICIPANTE	10/1/2015
48575	FEMININO	12/5/1946	70	ATIVO	PARTICIPANTE	5/14/2015
52651	FEMININO	4/28/1953	64	ATIVO	PARTICIPANTE	12/28/2015
46314	FEMININO	9/9/1943	73	ATIVO	PARTICIPANTE	9/4/2015
46741	MASCULINO	12/21/1938	78	ATIVO	DESISTENTE	1/16/2015
50422	MASCULINO	6/11/1950	66	ATIVO	PARTICIPANTE	10/16/2015
63903	FEMININO	6/3/1917	100	ATIVO	PARTICIPANTE	6/24/2015
134970	MASCULINO	2/5/1952	65	ATIVO	PARTICIPANTE	5/20/2015
74291	FEMININO	4/23/1940	77	ATIVO	PARTICIPANTE	2/27/2015
54362	FEMININO	6/12/1954	62	ATIVO	PARTICIPANTE	4/14/2015
76345	FEMININO	8/24/1932	84	ATIVO	PARTICIPANTE	6/19/2015
90931	FEMININO	8/22/1932	84	ATIVO	PARTICIPANTE	8/17/2015
55998	MASCULINO	3/2/1942	75	ATIVO	PARTICIPANTE	2/13/2015

49594	FEMININO	8/16/1938	78	ATIVO	PARTICIPANTE	2/4/2015
42606	MASCULINO	3/17/1949	68	ATIVO	PARTICIPANTE	10/27/2015
71322	MASCULINO	8/27/1952	64	ATIVO	PARTICIPANTE	3/12/2015
49661	MASCULINO	7/4/1945	71	ATIVO	PARTICIPANTE	2/13/2015
65065	FEMININO	6/24/1934	82	ATIVO	PARTICIPANTE	1/31/2015
72562	FEMININO	3/25/1941	76	ATIVO	PARTICIPANTE	1/29/2015
64256	FEMININO	1/4/1925	92	ATIVO	PARTICIPANTE	2/6/2015
72940	FEMININO	6/7/1939	77	ATIVO	PARTICIPANTE	7/31/2015
46313	MASCULINO	9/20/1940	76	ATIVO	PARTICIPANTE	9/4/2015
59957	FEMININO	10/31/1927	89	ATIVO	PARTICIPANTE	11/10/2015
50190	MASCULINO	5/24/1938	79	ATIVO	PARTICIPANTE	5/13/2015
55992	MASCULINO	11/5/1948	68	ATIVO	PARTICIPANTE	9/30/2015
51315	MASCULINO	9/11/1938	78	ATIVO	PARTICIPANTE	8/25/2015
74510	FEMININO	7/8/1951	65	ATIVO	PARTICIPANTE	9/2/2015
70171	MASCULINO	3/27/1954	63	ATIVO	PARTICIPANTE	10/28/2015
47604	MASCULINO	4/4/1947	70	ATIVO	PARTICIPANTE	10/8/2015
62551	FEMININO	9/2/1943	73	ATIVO	PARTICIPANTE	1/20/2015
48499	FEMININO	11/3/1938	78	ATIVO	PARTICIPANTE	1/19/2015
87017	MASCULINO	4/1/1942	75	ATIVO	PARTICIPANTE	3/19/2015
53299	MASCULINO	11/7/1939	77	ATIVO	PARTICIPANTE	4/24/2015
47129	MASCULINO	6/26/1949	67	ATIVO	PARTICIPANTE	10/8/2015
50978	MASCULINO	9/15/1941	75	ATIVO	PARTICIPANTE	3/3/2015
52517	FEMININO	7/13/1946	70	ATIVO	PARTICIPANTE	1/5/2015
72421	MASCULINO	1/20/1946	71	ATIVO	PARTICIPANTE	3/20/2015
59711	MASCULINO	1/13/1951	66	ATIVO	PARTICIPANTE	6/26/2015
51338	MASCULINO	5/3/1944	73	ATIVO	PARTICIPANTE	9/21/2015
47643	FEMININO	12/25/1944	72	ATIVO	PARTICIPANTE	3/13/2015
45192	FEMININO	8/4/1939	77	ATIVO	PARTICIPANTE	7/1/2015
70240	FEMININO	4/2/1935	82	ATIVO	PARTICIPANTE	2/23/2015
49236	FEMININO	12/15/1948	68	ATIVO	PARTICIPANTE	5/14/2015
104113	MASCULINO	4/5/1929	88	ATIVO	PARTICIPANTE	2/12/2015
43244	MASCULINO	8/20/1941	75	ATIVO	PARTICIPANTE	3/25/2015
66577	FEMININO	7/24/1948	68	ATIVO	PARTICIPANTE	11/7/2015
99030	MASCULINO	9/29/1935	81	ATIVO	PARTICIPANTE	1/12/2015
46799	MASCULINO	6/15/1939	77	ATIVO	PARTICIPANTE	1/23/2015
60353	MASCULINO	11/8/1940	76	ATIVO	PARTICIPANTE	3/20/2015
71778	FEMININO	3/28/1931	86	ATIVO	PARTICIPANTE	11/27/2015
65124	FEMININO	4/2/1938	79	ATIVO	PARTICIPANTE	6/23/2015
109824	FEMININO	8/9/1921	95	ATIVO	PARTICIPANTE	4/6/2015
45445	MASCULINO	6/14/1946	70	ATIVO	PARTICIPANTE	2/19/2015
51700	MASCULINO	3/17/1947	70	ATIVO	PARTICIPANTE	1/21/2015
47219	MASCULINO	9/7/1949	67	ATIVO	PARTICIPANTE	9/28/2015
59277	MASCULINO	10/13/1940	76	ATIVO	PARTICIPANTE	2/12/2015
50719	MASCULINO	2/24/1945	72	ATIVO	PARTICIPANTE	10/16/2015
46822	MASCULINO	11/15/1937	79	ATIVO	PARTICIPANTE	1/9/2015
43063	MASCULINO	7/26/1945	71	ATIVO	PARTICIPANTE	6/30/2015
50993	MASCULINO	3/6/1948	69	ATIVO	PARTICIPANTE	10/6/2015
96347	FEMININO	12/19/1950	66	ATIVO	PARTICIPANTE	7/16/2015
79474	FEMININO	8/13/1956	60	ATIVO	PARTICIPANTE	11/19/2015
58806	FEMININO	3/16/1947	70	ATIVO	PARTICIPANTE	7/24/2015
46954	MASCULINO	12/2/1947	69	ATIVO	PARTICIPANTE	4/9/2015
46236	FEMININO	2/28/1930	87	INATIVO	OBITO	5/7/2015
94524	FEMININO	6/7/1935	81	ATIVO	PARTICIPANTE	7/17/2015
47482	MASCULINO	12/23/1945	71	ATIVO	PARTICIPANTE	3/9/2015
44654	FEMININO	7/30/1946	70	ATIVO	PARTICIPANTE	5/22/2015
56629	MASCULINO	11/15/1950	66	ATIVO	PARTICIPANTE	12/8/2015

60885	FEMININO	3/9/1927	90	INATIVO	EXCLUIDO DO PLANO	9/26/2015
101588	MASCULINO	1/10/1938	79	ATIVO	PARTICIPANTE	7/9/2015

DT_SAIDA	MEDICO	CLASSIFICACAO	CARDIOPATIA	ICC	ANGINA	INFARTO	CIRURGIA
9/17/2016	JULIANA	IDOSO FRAGIL	SIM		SIM		
	GABRIELLA	IDOSO ROBUSTO	SIM	SIM			ARRITMIA
11/12/2016	RONAN	EM RISCO DE FRAGILIZACAO	SIM	SIM			
11/14/2016	JAMILLE	EM RISCO DE FRAGILIZACAO	SIM			SIM	
	FLAVIA	EM RISCO DE FRAGILIZACAO	SIM			SIM	
	GABRIELLA	EM RISCO DE FRAGILIZACAO	SIM			SIM	
	JAMILLE	IDOSO ROBUSTO	SIM			SIM	
	FLAVIA	IDOSO FRAGIL	SIM	SIM			
1/0/1900	GABRIELLA	IDOSO FRAGIL	SIM	SIM			
	NICOLAS	EM RISCO DE FRAGILIZACAO	SIM			SIM	
	RONAN	IDOSO FRAGIL	SIM			SIM	
	LETICIA	IDOSO ROBUSTO	SIM			SIM	
	JULIANA	IDOSO ROBUSTO	SIM			SIM	
	FLAVIA	EM RISCO DE FRAGILIZACAO	SIM			SIM	
	JAMILLE	IDOSO ROBUSTO	SIM		SIM		CATE, FA
7/13/2016	JULIANA	IDOSO ROBUSTO	SIM			SIM	
	GABRIELLA	EM RISCO DE FRAGILIZACAO	SIM			SIM	
	CAMILA	IDOSO FRAGIL	SIM			SIM	
	JULIANA	IDOSO ROBUSTO	SIM			SIM	
	GABRIELLA	IDOSO ROBUSTO	SIM			SIM	
	CAMILA	EM RISCO DE FRAGILIZACAO	SIM	SIM			
	LETICIA	IDOSO ROBUSTO	SIM			SIM	
11/16/2016	JAMILLE	EM RISCO DE FRAGILIZACAO	SIM			IAM1997	
1/0/1900	FLAVIA	IDOSO FRAGIL	SIM	SIM		SIM	
	JAMILLE	EM RISCO DE FRAGILIZACAO	SIM	SIM			
	JAMILLE	IDOSO ROBUSTO	SIM			SIM	ANGIOPLASTIA 2002
	GABRIELLA	EM RISCO DE FRAGILIZACAO	SIM	SIM			
	LIV	IDOSO ROBUSTO	SIM			IAM 2012	
	FLAVIA	IDOSO ROBUSTO	SIM	SIM			
	CAMILA	EM RISCO DE FRAGILIZACAO	SIM	SIM			
	LETICIA	IDOSO ROBUSTO	SIM			IAM 2010	
	FLAVIA	EM RISCO DE FRAGILIZACAO	SIM			SIM	
	JAMILLE	IDOSO ROBUSTO	SIM			SIM	
	GABRIELLA	IDOSO ROBUSTO	SIM			SIM	
	JAMILLE	EM RISCO DE FRAGILIZACAO	SIM		SIM		
	JULIANA	EM RISCO DE FRAGILIZACAO	SIM			SIM	
	JAMILLE	EM RISCO DE FRAGILIZACAO	SIM			IAM 1997	
	LETICIA	IDOSO ROBUSTO	SIM			SIM	
11/4/2016	GABRIELLA	EM RISCO DE FRAGILIZACAO	SIM			SIM	
	FLAVIA	EM RISCO DE FRAGILIZACAO	SIM			SIM	
	JULIANA	IDOSO ROBUSTO	SIM			SIM	
2/27/2016	FLAVIA	IDOSO ROBUSTO	SIM			SIM	
	GABRIELLA	IDOSO ROBUSTO	SIM			SIM	
	FLAVIA	EM RISCO DE FRAGILIZACAO	SIM			SIM	
	CAMILA	IDOSO ROBUSTO	SIM			SIM	
11/30/2016	LETICIA	IDOSO ROBUSTO	SIM			SIM	
	FLAVIA	EM RISCO DE FRAGILIZACAO	SIM			SIM	
	FLAVIA	EM RISCO DE FRAGILIZACAO	SIM			SIM	
	FLAVIA	IDOSO ROBUSTO	SIM			SIM	
	FLAVIA	IDOSO ROBUSTO	SIM			SIM	
	CAMILA	EM RISCO DE FRAGILIZACAO	SIM			SIM	
	RONAN	IDOSO ROBUSTO	SIM			SIM	
	JAMILLE	IDOSO ROBUSTO	SIM	SIM			

	LETICIA	IDOSO ROBUSTO	SIM			SIM	
	FLAVIA	EM RISCO DE FRAGILIZACAO	SIM			SIM	
	LETICIA	IDOSO ROBUSTO	SIM			SIM	
	LIV	IDOSO ROBUSTO	SIM			SIM	
	JULIANA	EM RISCO DE FRAGILIZACAO	SIM		SIM		
	LETICIA	IDOSO ROBUSTO	SIM	SIM			
	LETICIA	IDOSO ROBUSTO	SIM	SIM			
	FLAVIA	EM RISCO DE FRAGILIZACAO	SIM			IAM 2004	
	JAMILLE	IDOSO ROBUSTO	SIM	SIM	SIM		
	FLAVIA	EM RISCO DE FRAGILIZACAO	SIM			SIM	
	JULIANA	IDOSO FRAGIL	SIM	SIM			
	JAMILLE	EM RISCO DE FRAGILIZACAO	SIM			IAM 2011	CAT
	FLAVIA	IDOSO ROBUSTO	SIM			IAM 2009	
	JAMILLE	IDOSO ROBUSTO	SIM			SIM	
	LIV	IDOSO ROBUSTO	SIM			SIM	
	FLAVIA	IDOSO ROBUSTO	SIM			SIM	
	LETICIA	EM RISCO DE FRAGILIZACAO	SIM	SIM		SIM	
	LETICIA	IDOSO ROBUSTO	SIM			SIM	
	JAMILLE	EM RISCO DE FRAGILIZACAO	SIM			IAM 2006	
	JULIANA	EM RISCO DE FRAGILIZACAO	SIM			SIM	
	JAMILLE	IDOSO ROBUSTO	SIM			IAM 2008	
	GABRIELLA	EM RISCO DE FRAGILIZACAO	SIM			SIM	
	FLAVIA	EM RISCO DE FRAGILIZACAO	SIM			SIM	
	JAMILLE	EM RISCO DE FRAGILIZACAO	SIM			SIM	
	JULIANA	IDOSO ROBUSTO	SIM			SIM	
	JAMILLE	IDOSO ROBUSTO	SIM			SIM	
	FLAVIA	IDOSO ROBUSTO	SIM		SIM		
1/12/2017	FLAVIA	EM RISCO DE FRAGILIZACAO	SIM			SIM	
	LIV	IDOSO ROBUSTO	SIM			SIM	
	CAMILA	EM RISCO DE FRAGILIZACAO	SIM			SIM	
	JULIANA	EM RISCO DE FRAGILIZACAO	SIM		SIM		
	JULIANA	IDOSO ROBUSTO	SIM			IAM 2013	
	RONAN	IDOSO ROBUSTO	SIM			SIM	
	FLAVIA	IDOSO ROBUSTO	SIM			SIM	
	FLAVIA	IDOSO ROBUSTO	SIM			SIM	
	GABRIELLA	IDOSO ROBUSTO	SIM			SIM	
	FLAVIA	IDOSO ROBUSTO	SIM			SIM	
	FLAVIA	IDOSO ROBUSTO	SIM			SIM	
	GABRIELLA	IDOSO ROBUSTO	SIM			SIM	
	GABRIELLA	IDOSO ROBUSTO	SIM			SIM	
	LETICIA	EM RISCO DE FRAGILIZACAO	SIM			SIM	
	FLAVIA	EM RISCO DE FRAGILIZACAO	SIM			IAM 2005	
	RONAN	IDOSO ROBUSTO	SIM			SIM	
	JAMILLE	EM RISCO DE FRAGILIZACAO	SIM			SIM	
	GABRIELLA	EM RISCO DE FRAGILIZACAO	SIM			SIM	
	NICOLAS	IDOSO ROBUSTO	SIM			SIM	
	RONAN	EM RISCO DE FRAGILIZACAO	SIM			SIM	
	CAMILA	IDOSO ROBUSTO	SIM	SIM		SIM	
	LETICIA	IDOSO ROBUSTO	SIM			SIM	
	RONAN	IDOSO FRAGIL	SIM	SIM			
	NICOLAS	EM RISCO DE FRAGILIZACAO	SIM			SIM	NEO MET
	LETICIA	IDOSO FRAGIL	SIM			SIM	
	JULIANA	EM RISCO DE FRAGILIZACAO	SIM	SIM			
	LIV	IDOSO ROBUSTO	SIM			SIM	
	GABRIELLA	EM RISCO DE FRAGILIZACAO	SIM			SIM	
	RONAN	IDOSO ROBUSTO	SIM			SIM	

	GABRIELLA	IDOSO FRAGIL	SIM	SIM			
	LIV	EM RISCO DE FRAGILIZACAO	SIM			SIM	
	CAMILA	EM RISCO DE FRAGILIZACAO	SIM			SIM	
	JAMILLE	EM RISCO DE FRAGILIZACAO	SIM			SIM	
	NICOLAS	IDOSO ROBUSTO	SIM	SIM		SIM	
	LETICIA	IDOSO FRAGIL	SIM			SIM	
	RONAN	IDOSO ROBUSTO	SIM			SIM	
	GABRIELLA	IDOSO ROBUSTO	SIM			SIM	
	JULIANA	EM RISCO DE FRAGILIZACAO	SIM			SIM	
	RONAN	EM RISCO DE FRAGILIZACAO	SIM			SIM	
	LIV	IDOSO ROBUSTO	SIM			SIM	
	FLAVIA	IDOSO ROBUSTO	SIM			IAM 2006	
	FLAVIA	EM RISCO DE FRAGILIZACAO	SIM			SIM	
	JULIANA	EM RISCO DE FRAGILIZACAO	SIM			SIM	
	FLAVIA	IDOSO ROBUSTO	SIM			SIM	
	FLAVIA	IDOSO ROBUSTO	SIM			SIM	
	CAMILA	EM RISCO DE FRAGILIZACAO	SIM			SIM	
	FLAVIA	IDOSO ROBUSTO	SIM			IAM 2005	
	LETICIA	IDOSO ROBUSTO	SIM	SIM		SIM	
	GABRIELLA	IDOSO FRAGIL	SIM			SIM	
	JAMILLE	EM RISCO DE FRAGILIZACAO	SIM	SIM			
	FLAVIA	EM RISCO DE FRAGILIZACAO	SIM			SIM	
	JAMILLE	EM RISCO DE FRAGILIZACAO	SIM			SIM	
	JULIANA	IDOSO ROBUSTO	SIM			IAM 2007	
	GABRIELLA	EM RISCO DE FRAGILIZACAO	SIM			SIM	
	RONAN	IDOSO ROBUSTO	SIM			SIM	
	LETICIA	EM RISCO DE FRAGILIZACAO	SIM		SIM		
	FLAVIA	IDOSO ROBUSTO	SIM			IAM 2013	
	FLAVIA	EM RISCO DE FRAGILIZACAO	SIM			SIM	
	FLAVIA	IDOSO ROBUSTO	SIM	SIM			
	FLAVIA	EM RISCO DE FRAGILIZACAO	SIM		SIM		
	LETICIA	EM RISCO DE FRAGILIZACAO	SIM			SIM	
2/29/2016	GABRIELLA	IDOSO ROBUSTO	SIM			SIM	
	NICOLAS	EM RISCO DE FRAGILIZACAO	SIM			SIM	
	JAMILLE	IDOSO ROBUSTO	SIM		SIM		
	GABRIELLA	IDOSO ROBUSTO	SIM			SIM	
	JAMILLE	IDOSO FRAGIL	SIM		SIM		
	JULIANA	IDOSO ROBUSTO	SIM			SIM	
	JULIANA	IDOSO ROBUSTO	SIM			SIM	
	JAMILLE	IDOSO ROBUSTO	SIM			SIM	
	JULIANA	EM RISCO DE FRAGILIZACAO	SIM			SIM	
	GABRIELLA	IDOSO ROBUSTO	SIM			SIM	
	LIV	EM RISCO DE FRAGILIZACAO	SIM	SIM		SIM	
	JAMILLE	IDOSO ROBUSTO	SIM		SIM		
	JAMILLE	IDOSO ROBUSTO	SIM			SIM	
	NICOLAS	EM RISCO DE FRAGILIZACAO	SIM			SIM	
	LETICIA	IDOSO ROBUSTO	SIM	SIM			
4/17/2017	JULIANA	IDOSO ROBUSTO	SIM			SIM	
	JAMILLE	IDOSO ROBUSTO	SIM			SIM	
	JULIANA	IDOSO FRAGIL	SIM	SIM			FA
	RONAN	IDOSO ROBUSTO	SIM			SIM	
	CAMILA	EM RISCO DE FRAGILIZACAO	SIM			SIM	
	RONAN	EM RISCO DE FRAGILIZACAO	SIM	SIM			
	LIV	EM RISCO DE FRAGILIZACAO	SIM			SIM	
	GABRIELLA	EM RISCO DE FRAGILIZACAO	SIM			SIM	
	GABRIELLA	IDOSO ROBUSTO	SIM			SIM	

	JAMILLE	IDOSO ROBUSTO	SIM			SIM	
	FLAVIA	IDOSO ROBUSTO	SIM			IAM 2013	
	RONAN	IDOSO ROBUSTO	SIM			SIM	
	FLAVIA	IDOSO ROBUSTO	SIM			SIM	
	GABRIELLA	EM RISCO DE FRAGILIZACAO	SIM	SIM			
	FLAVIA	EM RISCO DE FRAGILIZACAO	SIM			SIM	
	LETICIA	IDOSO FRAGIL	SIM		SIM		
	GABRIELLA	IDOSO FRAGIL	SIM			SIM	
	LETICIA	IDOSO ROBUSTO	SIM	SIM			
	FLAVIA	IDOSO FRAGIL	SIM			SIM	
	JAMILLE	IDOSO ROBUSTO	SIM	SIM			
	FLAVIA	IDOSO ROBUSTO	SIM			SIM	
	FLAVIA	IDOSO ROBUSTO	SIM			SIM	
	JAMILLE	EM RISCO DE FRAGILIZACAO	SIM			SIM	
	GABRIELLA	EM RISCO DE FRAGILIZACAO	SIM			SIM	
	LETICIA	IDOSO ROBUSTO	SIM			SIM 2009	
	CAMILA	IDOSO ROBUSTO	SIM		SIM		
	GABRIELLA	EM RISCO DE FRAGILIZACAO	SIM		SIM		
	RONAN	IDOSO FRAGIL	SIM			SIM	
	NICOLAS	IDOSO ROBUSTO	SIM			SIM	
	FLAVIA	IDOSO ROBUSTO	SIM		SIM		
	RONAN	IDOSO ROBUSTO	SIM			SIM	
	LETICIA	EM RISCO DE FRAGILIZACAO	SIM			SIM	
	CAMILA	IDOSO ROBUSTO	SIM			SIM	
	FLAVIA	EM RISCO DE FRAGILIZACAO	SIM		SIM		
	JULIANA	IDOSO ROBUSTO	SIM			SIM	
	GABRIELLA	IDOSO ROBUSTO	SIM			SIM	
	LETICIA	EM RISCO DE FRAGILIZACAO	SIM	SIM			
	GABRIELLA	IDOSO ROBUSTO	SIM	SIM	SIM	SIM	
	LETICIA	EM RISCO DE FRAGILIZACAO	SIM			IAM 2001	
	GABRIELLA	EM RISCO DE FRAGILIZACAO	SIM	SIM			
	FLAVIA	IDOSO ROBUSTO	SIM			IAM JAN/2015	
	JAMILLE	EM RISCO DE FRAGILIZACAO	SIM			SIM	
	RONAN	IDOSO ROBUSTO	SIM			SIM	
	CAMILA	EM RISCO DE FRAGILIZACAO	SIM	SIM			
	NICOLAS	IDOSO FRAGIL	SIM			SIM	
	GABRIELLA	IDOSO ROBUSTO	SIM			SIM	
	GABRIELLA	IDOSO ROBUSTO	SIM	SIM			
	GABRIELLA	IDOSO FRAGIL	SIM			SIM	
	JAMILLE	IDOSO ROBUSTO	SIM			IAM 2013	
	RONAN	IDOSO ROBUSTO	SIM	SIM			
	FLAVIA	IDOSO ROBUSTO	SIM			SIM	
	FLAVIA	IDOSO ROBUSTO	SIM			SIM	
	JULIANA	EM RISCO DE FRAGILIZACAO	SIM			SIM	
	JAMILLE	IDOSO FRAGIL	SIM	SIM		SIM	
	FLAVIA	IDOSO ROBUSTO	SIM			IAM 2004	
	JAMILLE	IDOSO ROBUSTO	SIM			SIM	
	NICOLAS	EM RISCO DE FRAGILIZACAO	SIM			SIM	
	LETICIA	IDOSO ROBUSTO	SIM			SIM	
	JULIANA	EM RISCO DE FRAGILIZACAO	SIM			SIM	
	FLAVIA	IDOSO FRAGIL	SIM		SIM		ANGIOPLASTIA 2008
2/28/2017	JAMILLE	IDOSO FRAGIL	SIM	SIM			
	JULIANA	IDOSO FRAGIL	SIM	SIM			
	FLAVIA	IDOSO ROBUSTO	SIM			SIM	
	GABRIELLA	EM RISCO DE FRAGILIZACAO	SIM		SIM		
	CAMILA	IDOSO ROBUSTO	SIM			SIM	

1/31/2017	FLAVIA	EM RISCO DE FRAGILIZACAO	SIM			SIM	
	RONAN	IDOSO FRAGIL	SIM	SIM			

PESO	ALTURA	IMC	CLASSIFICACAO IMC > 60 ANOS	TABAGISMO	RASTREIO CID	CUSTO TOTAL ANTES	CUSTO TOTAL DEPOIS
-	-		SOBREPESO	NÃO		R$ 3,643.53	R$ 44,789.57
82.8	1.72	27.99	SOBREPESO	NÃO		R$ -	R$ 40,932.83
75	1.6	29.30	SOBREPESO	-		R$ 2,432.50	R$ 42,459.44
68.25	1.63	25.69	NORMAL	-		R$ 583.71	R$ 33,138.85
85.5	1.6	33.40	SOBREPESO	NÃO		R$ 58.00	R$ 31,378.33
-	-	#VALUE!		-		R$ 756.20	R$ 29,323.71
89.6	1.65	32.91	SOBREPESO	NÃO		R$ 1,273.86	R$ 26,371.24
61.15	1.42	30.33	SOBREPESO	NÃO		R$ 1,103.97	R$ 22,872.86
69.4	1.59	27.45	SOBREPESO	-		R$ 5,950.42	R$ 24,995.13
74.2	1.65	27.25	SOBREPESO	NÃO		R$ 2,699.95	R$ 20,774.96
104	1.55	43.29	SOBREPESO	NÃO		R$ 24,954.87	R$ 41,305.87
76	1.59	30.06	SOBREPESO	NÃO		R$ 864.76	R$ 15,100.77
66.5	1.66	24.13	NORMAL	SIM		R$ 2,598.42	R$ 15,685.18
70.8	1.56	29.09	SOBREPESO	NÃO		R$ 3,044.85	R$ 14,879.91
97.7	1.65	35.89	SOBREPESO	NÃO		R$ 4,320.72	R$ 14,374.23
38	1.3	22.49	NORMAL	NÃO		R$ 594.61	R$ 8,443.75
81.15	1.54	34.22	SOBREPESO	NÃO		R$ 1,123.47	R$ 8,823.35
87.3	1.69	30.57	SOBREPESO	NÃO		R$ -	R$ 7,139.31
78	1.69	27.31	SOBREPESO	NÃO		R$ 3,492.73	R$ 10,271.60
81.75	1.69	28.62	SOBREPESO	NÃO		R$ 116.00	R$ 6,672.07
67.6	1.46	31.71	SOBREPESO	NÃO		R$ 5,917.14	R$ 12,424.04
60.9	1.67	21.84	BAIXO PESO	SIM		R$ 923.30	R$ 7,296.81
1.72	83.8	0.00	BAIXO PESO	NÃO		R$ 3,347.57	R$ 9,002.58
74.75	1.65	27.46	SOBREPESO	NÃO		R$ 5,492.86	R$ 10,976.29
64	1.52	27.70	SOBREPESO	NÃO		R$ 667.27	R$ 6,090.90
-	-	#VALUE!	#VALUE!	-		R$ 1,052.16	R$ 6,319.15
-	-	#VALUE!		-		R$ 2,185.46	R$ 7,296.87
95.6	1.62	36.43	SOBREPESO	NÃO		R$ -	R$ 4,851.05
86.35	1.58	34.59	SOBREPESO	NÃO		R$ 481.29	R$ 5,322.13
72.8	1.57	29.53	SOBREPESO	-		R$ -	R$ 4,463.99
77.15	1.59	30.52	SOBREPESO	-		R$ 845.63	R$ 5,253.62
92.9	1.55	38.67	SOBREPESO	NÃO		R$ 1,624.00	R$ 6,018.91
67	1.72	22.65	NORMAL	NÃO		R$ 785.36	R$ 5,108.62
88.3	1.73	29.50	SOBREPESO	NÃO		R$ 1,124.38	R$ 4,841.35
65.1	1.52	28.18	SOBREPESO	SIM		R$ 1,022.34	R$ 4,694.47
57.6	1.43	28.17	SOBREPESO	NÃO		R$ 126.25	R$ 3,475.12
58.4	1.5	25.96	NORMAL	NÃO		R$ 2,099.80	R$ 4,969.05
63.85	1.59	25.26	NORMAL	-		R$ 1,564.24	R$ 4,429.79
79.5	1.7	27.51	SOBREPESO	NÃO		R$ -	R$ 2,646.51
62	1.55	25.81	NORMAL	NÃO		R$ 3,558.72	R$ 6,185.24
68	1.64	25.28	NORMAL	NÃO		R$ 200.99	R$ 2,395.02
86.4	1.62	32.92	SOBREPESO	NÃO		R$ 2,372.04	R$ 4,540.20
68.9	1.59	27.25	SOBREPESO	NÃO		R$ 130.77	R$ 2,295.10
84.2	1.56	34.60	SOBREPESO	NAOEX		R$ 393.29	R$ 2,490.21
97	1.66	#VALUE!	#VALUE!	NÃO		R$ 550.85	R$ 2,474.69
80.65	1.65	29.62	SOBREPESO	-		R$ 102.92	R$ 2,019.87
85	1.68	30.12	SOBREPESO	NÃO		R$ 1,845.98	R$ 3,720.12
83.3	1.54	35.12	SOBREPESO	NÃO		R$ 1,063.82	R$ 2,927.69
100.5	1.68	35.61	SOBREPESO	NÃO		R$ 669.93	R$ 2,500.30
93.1	1.68	32.99	SOBREPESO	NÃO		R$ 58.00	R$ 1,814.90
67	1.62	25.53	NORMAL	NÃO		R$ 113.00	R$ 1,866.35
57	1.57	23.12	NORMAL	NÃO		R$ -	R$ 1,736.81
89.9	1.76	29.02	SOBREPESO	NÃO		R$ 385.97	R$ 1,929.35

60.9	1.68	21.58	BAIXO PESO	SIM		R$ 953.54	R$ 2,464.35
76.7	1.73	25.63	NORMAL	NÃO		R$ 521.37	R$ 1,974.94
66.6	1.56	27.37	SOBREPESO	NÃO		R$ 1,940.89	R$ 3,280.35
79.4	1.7	27.47	SOBREPESO	-		R$ -	R$ 1,297.00
51	1.51	22.37	NORMAL	NÃO		R$ 1,854.83	R$ 3,138.04
85.4	1.68	30.26	SOBREPESO	NÃO		R$ 194.24	R$ 1,411.69
65.05	1.55	27.08	SOBREPESO	NÃO		R$ 359.31	R$ 1,565.12
101.45	1.65	37.26	SOBREPESO	NAOEX	I209	R$ 5,097.90	R$ 6,204.80
102250	1.64	38016.81	SOBREPESO	NÃO		R$ 1,128.55	R$ 2,194.52
66.4	1.58	26.60	NORMAL	NÃO		R$ 232.00	R$ 1,127.27
91.2	1.78	28.78	SOBREPESO	NÃO		R$ 3,574.40	R$ 4,458.06
69	1.6	26.95	FALSE	NÃO		R$ 754.51	R$ 1,573.01
133.1	1.77	42.48	SOBREPESO	-		R$ -	R$ 795.69
83.1	1.63	31.28	SOBREPESO	SIM		R$ -	R$ 781.85
-	-	#VALUE!	#VALUE!	NÃO		R$ -	R$ 735.42
77.5	1.68	27.46	SOBREPESO	NÃO		R$ 3,377.73	R$ 4,059.15
75.65	1.59	29.92	SOBREPESO	NÃO		R$ -	R$ 651.84
89.6	1.6	35.00	SOBREPESO	-		R$ -	R$ 631.90
86.50	1.66	#VALUE!	#VALUE!	NÃO		R$ 253.24	R$ 884.78
57	1.41	28.67	SOBREPESO	NÃO		R$ 3,997.91	R$ 4,594.23
86.1	1.61	33.22	SOBREPESO	NÃO		R$ 141.56	R$ 723.31
80.45	1.55	33.49	SOBREPESO	NÃO		R$ 4,125.35	R$ 4,703.52
45.2	1.5	20.09	BAIXO PESO	NÃO		R$ 298.18	R$ 873.35
72	1.53	30.76	SOBREPESO	NÃO		R$ -	R$ 560.85
67	1.6	26.17	NORMAL	NÃO		R$ 1,284.87	R$ 1,828.60
77.8	1.67	27.90	SOBREPESO	NÃO		R$ 199.26	R$ 689.23
71	1.62	27.05	SOBREPESO	NÃO		R$ 1,342.18	R$ 1,826.04
68	1.68	24.09	NORMAL	NÃO		R$ -	R$ 445.25
70	1.71	23.94	NORMAL	NÃO		R$ 4,881.80	R$ 5,310.86
78.9	1.75	25.76	NORMAL	SIM		R$ 1,610.40	R$ 2,017.04
70	1.52	30.30	SOBREPESO	-		R$ 324.36	R$ 723.13
-	-	#VALUE!	#VALUE!	NÃO		R$ 798.39	R$ 1,111.87
70	1.65	25.71	NORMAL	NÃO		R$ 890.22	R$ 1,182.70
65	1.6	25.39	NORMAL	NÃO		R$ -	R$ 244.03
73	1.58	29.24	SOBREPESO	NÃO		R$ 195.28	R$ 433.70
85.8	1.64	31.90	SOBREPESO	SIM		R$ -	R$ 186.78
47.85	1.6	18.69	BAIXO PESO	SIM		R$ -	R$ 174.05
79	1.69	27.66	SOBREPESO	NÃO		R$ 1,818.77	R$ 1,982.86
66	1.65	24.24	NORMAL	SIM		R$ -	R$ 156.10
78.95	1.77	25.20	NORMAL	-		R$ -	R$ 150.15
80.25	1.56	32.98	SOBREPESO	NÃO		R$ 456.25	R$ 602.81
60.75	1.48	27.73	SOBREPESO	NÃO		R$ 1,031.30	R$ 1,143.11
78	1.6	30.47	SOBREPESO	NÃO		R$ -	R$ 70.01
83.75	1.6	32.71	SOBREPESO	NÃO		R$ 270.91	R$ 318.02
57.6	1.66	20.90	BAIXO PESO	NÃO		R$ -	R$ 33.30
70.75	1.56	29.07	SOBREPESO	-		R$ -	R$ 32.64
65	1.72	21.97	FALSE	NÃO		R$ -	R$ 32.64
108.6	1.64	40.38	SOBREPESO	NÃO		R$ -	R$ 32.62
105.95	1.69	37.10	SOBREPESO	NÃO		R$ -	R$ 32.60
82.1	1.52	35.53	SOBREPESO	SIM		R$ -	R$ 10.97
-	-	#VALUE!		-		R$ -	R$ 10.91
81.95	1.67	29.38	SOBREPESO	NÃO		R$ -	R$ 10.91
90	1.75	29.39	SOBREPESO	SIM		R$ -	R$ 10.88
80.2	1.62	30.56	SOBREPESO	NÃO		R$ -	R$ 10.87
76.2	1.66	27.65	SOBREPESO	NÃO		R$ -	R$ 0.14
85.5	1.73	28.57	SOBREPESO	NÃO		R$ -	R$ 0.05

53.1	1.53	22.68	NORMAL	NAOEX		R$	-	R$	0.05
86.3	1.64	32.09	SOBREPESO	SIM		R$	-	R$	0.04
93.6	1.72	31.64	SOBREPESO	NÃO		R$	-	R$	0.04
52.45	1.57	21.28	BAIXO PESO	NÃO		R$	-	R$	0.03
75	1.65	27.55	SOBREPESO	NÃO		R$	0.01	R$	0.04
81.35	1.53	34.75	SOBREPESO	-		R$	-	R$	0.03
65	1.61	25.08	NORMAL	NÃO		R$	-	R$	0.03
66.4	1.71	22.71	NORMAL	NÃO		R$	-	R$	0.02
-	-	#VALUE!	#VALUE!	SIM		R$	-	R$	0.01
52	1.57	21.10	BAIXO PESO	NÃO		R$	-	R$	0.01
-	-	#VALUE!	#VALUE!	-		R$	-	R$	0.01
62.5	1.65	22.96	NORMAL	NÃO		R$	-	R$	0.01
80.4	1.7	27.82	SOBREPESO	NÃO		R$	413.90	R$	404.67
91	1.61	35.11	SOBREPESO	-		R$	393.76	R$	364.49
70.15	1.59	27.75	SOBREPESO	NÃO		R$	603.97	R$	511.77
62.6	1.57	25.40	NORMAL	NÃO		R$	229.53	R$	124.76
95.5	1.68	33.84	SOBREPESO	NÃO		R$	1,796.47	R$	1,662.65
81	1.65	29.75	SOBREPESO	NÃO		R$	825.03	R$	672.54
78.8	1.75	25.73	NORMAL	SIM		R$	1,927.56	R$	1,770.52
51.7	1.5	22.98	NORMAL	NÃO		R$	160.33	R$	0.05
73	1.62	27.82	SOBREPESO	NÃO		R$	771.77	R$	607.48
78.3	1.68	27.74	SOBREPESO	NÃO		R$	3,825.47	R$	3,660.71
62.7	1.57	25.44	NORMAL	NÃO		R$	470.76	R$	300.05
74	1.54	31.20	SOBREPESO	NÃO		R$	483.11	R$	274.82
67.8	1.49	30.54	SOBREPESO	NÃO		R$	941.13	R$	667.18
95.6	1.67	34.28	SOBREPESO	NÃO		R$	348.58	R$	65.96
69.3	1.6	27.07	SOBREPESO	NAOEX		R$	1,783.13	R$	1,448.68
83.8	1.64	31.16	SOBREPESO	NÃO		R$	1,078.65	R$	736.66
49.7	1.41	25.00	NORMAL	NÃO		R$	753.04	R$	359.14
70.6	1.65	25.93	NORMAL	NÃO		R$	1,099.42	R$	660.56
70.1	1.48	32.00	SOBREPESO	NÃO		R$	759.96	R$	297.56
50.35	1.56	20.69	BAIXO PESO	NÃO		R$	2,478.33	R$	2,008.48
68.85	1.67	24.69	NORMAL	NÃO		R$	705.18	R$	211.63
77.65	1.65	28.52	SOBREPESO	NÃO		R$	1,028.16	R$	500.52
88.45	1.69	30.97	SOBREPESO	NÃO		R$	919.03	R$	378.22
-	-	#VALUE!	#VALUE!	-		R$	1,856.04	R$	1,310.97
95	1.65	34.89	SOBREPESO	-		R$	1,100.82	R$	429.69
94	1.75	30.69	SOBREPESO	NÃO		R$	1,089.88	R$	406.55
72	1.6	28.13	SOBREPESO	NÃO		R$	3,014.93	R$	2,322.50
69.35	1.67	24.87	NORMAL	NÃO		R$	1,081.31	R$	348.98
86.35	1.58	34.59	SOBREPESO	NÃO		R$	1,189.10	R$	301.35
65.95	1.65	24.22	NORMAL	NÃO		R$	1,901.44	R$	901.69
-	-	#VALUE!	#VALUE!	NÃO		R$	2,206.90	R$	1,109.79
90.35	1.67	32.40	SOBREPESO	NÃO		R$	1,357.27	R$	92.15
82.7	1.55	34.42	SOBREPESO	NÃO		R$	1,519.09	R$	216.38
70.55	1.6	27.56	SOBREPESO	NÃO		R$	2,571.55	R$	1,115.15
57.7	1.53	24.65	NORMAL	NÃO		R$	2,365.42	R$	903.32
66	1.59	26.11	NORMAL	NÃO		R$	1,749.76	R$	136.88
68.6	1.7	23.74	NORMAL	NÃO		R$	2,313.61	R$	628.34
58	1.45	27.59	SOBREPESO	NÃO		R$	1,886.52	R$	0.04
69.5	1.56	28.56	SOBREPESO	SIM		R$	22,525.17	R$	20,624.38
68.5	1.56	28.15	SOBREPESO	NÃO		R$	6,797.76	R$	4,879.50
87.85	1.64	32.66	SOBREPESO	NÃO		R$	2,642.89	R$	716.81
51.6	1.48	23.56	NORMAL	-		R$	2,666.96	R$	379.17
76.25	1.53	32.57	SOBREPESO	-		R$	3,524.37	R$	1,169.09
80	1.7	27.68	SOBREPESO	NÃO		R$	2,759.19	R$	318.49

68.75	1.5	30.56	SOBREPESO	NÃO		R$	2,820.67	R$	282.63
71.7	1.64	26.66	NORMAL	NÃO		R$	6,096.62	R$	3,448.67
90.4	1.6	35.31	SOBREPESO	NÃO		R$	2,765.96	R$	0.03
70	1.66	25.40	NORMAL	NÃO		R$	5,813.95	R$	2,695.81
-	-	#VALUE!		-		R$	3,412.06	R$	0.04
40.75	1.48	18.60	BAIXO PESO	SIM		R$	3,734.23	R$	207.33
56.7	1.45	26.97	FALSE	NÃO		R$	15,752.09	R$	11,988.58
65.7	1.45	31.25	SOBREPESO	NÃO		R$	5,817.44	R$	1,801.19
75.6	1.65	27.77	SOBREPESO	-		R$	9,702.19	R$	5,563.02
52.7	1.5	23.42	NORMAL	NÃO		R$	13,128.98	R$	8,983.72
77.9	1.71	26.64	NORMAL	NÃO		R$	7,183.56	R$	2,969.93
88.9	1.7	30.76	SOBREPESO	NÃO		R$	4,490.01	R$	271.76
70.7	1.61	27.28	SOBREPESO	NÃO		R$	5,743.94	R$	1,341.24
67	1.56	27.53	SOBREPESO	NÃO		R$	4,776.51	R$	148.28
105.8	1.64	39.34	SOBREPESO	NÃO		R$	5,140.40	R$	0.02
67.6	1.61	26.08	NORMAL	-		R$	7,727.69	R$	2,389.33
76.75	1.52	33.22	SOBREPESO	NÃO		R$	5,481.46	R$	35.16
61	1.48	27.85	SOBREPESO	-		R$	8,587.48	R$	3,036.87
83.6	1.63	31.47	SOBREPESO	NÃO		R$	5,584.75	R$	0.05
59.5	1.63	22.39	NORMAL	NÃO		R$	6,059.70	R$	359.52
84.65	1.74	27.96	SOBREPESO	NÃO		R$	6,163.22	R$	295.81
77.75	1.75	25.39	NORMAL	NÃO		R$	5,893.04	R$	0.06
98.05	1.53	41.89	SOBREPESO	NÃO	I209	R$	6,607.13	R$	602.94
63.9	1.55	26.60	NORMAL	NÃO		R$	6,580.53	R$	0.02
74.2	1.7	25.67	NORMAL	SIM		R$	6,614.02	R$	32.66
87	1.73	29.07	SOBREPESO	-		R$	7,283.93	R$	468.55
-	-	#VALUE!	#VALUE!	NÃO		R$	8,172.98	R$	1,010.21
98.95	1.53	42.27	SOBREPESO	NÃO		R$	7,513.18	R$	104.96
62.4	1.65	22.92	NORMAL	NÃO		R$	12,419.11	R$	4,855.43
82.05	1.68	29.07	SOBREPESO	-		R$	9,691.78	R$	0.03
60.95	1.65	22.39	NORMAL	-		R$	13,831.85	R$	4,109.25
78.75	1.59	31.15	SOBREPESO	-		R$	11,446.30	R$	903.06
70.6	1.53	30.16	SOBREPESO	NÃO		R$	14,127.58	R$	3,288.46
63.4	1.65	23.29	NORMAL	NÃO		R$	10,988.97	R$	10.95
76.4	1.58	30.60	SOBREPESO	NÃO		R$	14,358.52	R$	3,326.44
103.55	1.67	37.13	SOBREPESO	NÃO		R$	13,270.23	R$	1,722.80
45.95	1.45	21.85	BAIXO PESO	-		R$	19,296.40	R$	6,655.34
57.85	1.5	25.71	NORMAL	NÃO		R$	22,380.14	R$	9,275.16
-	-	#VALUE!	#VALUE!	NÃO		R$	17,091.89	R$	3,842.35
-	-	#VALUE!	#VALUE!	NÃO		R$	16,159.37	R$	1,386.13
73.5	1.7	25.43	NORMAL	NÃO		R$	15,928.20	R$	592.89
75.7	1.71	25.89	NORMAL	NÃO		R$	16,232.92	R$	889.11
73	1.59	28.88	SOBREPESO	NÃO		R$	18,540.66	R$	2,540.44
127.55	1.75	41.65	SOBREPESO	NÃO		R$	18,091.17	R$	0.08
73.4	1.67	26.32	NORMAL	NÃO		R$	21,456.70	R$	3,172.87
89.6	1.74	29.59	SOBREPESO	NÃO		R$	21,988.98	R$	978.55
90	1.72	30.42	SOBREPESO	NÃO		R$	24,667.33	R$	365.82
-	-	#VALUE!		-		R$	24,980.67	R$	0.09
99.3	1.5	44.13	SOBREPESO	SIM		R$	26,652.05	R$	651.50
87	1.52	37.66	SOBREPESO	NÃO		R$	28,551.05	R$	1,963.11
108	1.76	34.87	SOBREPESO	-		R$	29,716.23	R$	71.95
-	-	#VALUE!	#VALUE!	NÃO		R$	44,313.41	R$	13,255.95
87	1.62	33.15	SOBREPESO	NÃO		R$	34,827.13	R$	1,187.88
87.9	1.71	30.06	SOBREPESO	-		R$	34,407.47	R$	738.54
74.95	1.44	36.14	SOBREPESO	NÃO		R$	41,273.26	R$	5,344.53
89.2	1.73	29.80	SOBREPESO	NÃO		R$	41,258.42	R$	2,386.67

75.8	1.52	32.81	SOBREPESO	NÃO		R$ 46,128.10	R$ 80.35
105	1.66	38.10	SOBREPESO	NAOEX		R$ 60,733.41	R$ 5,485.16

Nº CONSULTAS PA ANTES	Nº CONSULTAS PA DEPOIS	CUSTO PA ANTES	CUSTO PA DEPOIS	Nº INTERNAÇÕES ANTES	Nº INTERNAÇÕES DEPOIS	CUSTO INTERNAÇÕES ANTES	CUSTO INTERNAÇÕES DEPOIS
1	2	R$ 73.00	R$ 161.00	1	2	R$ 3,562.66	R$ 43,990.33
					1		R$ 40,866.79
1		R$ 73.00		1	2	R$ 2,351.62	R$ 31,212.79
1	2	R$ 73.00	R$ 151.00		1		R$ 29,303.29
	3		R$ 229.00		1		R$ 30,141.28
	1		R$ 73.00		1		R$ 28,983.39
					2		R$ 26,371.22
2	4	R$ 146.00	R$ 307.00		3		R$ 20,125.72
				1	3	R$ 5,950.42	R$ 24,995.12
1	1	R$ 78.00	R$ 83.00	1	2	R$ 2,366.62	R$ 18,165.42
1		R$ 73.00		2	1	R$ 24,874.00	R$ 41,305.82
1	2	R$ 73.00	R$ 156.00		1		R$ 13,715.27
3	2	R$ 229.00	R$ 156.00		2		R$ 14,445.24
1	2	R$ 73.00	R$ 166.00	2	2	R$ 1,805.84	R$ 11,553.44
3	3	R$ 216.00	R$ 229.00	2	2	R$ 3,806.94	R$ 13,244.66
	1		R$ 78.00		1		R$ 8,024.51
1	1	R$ 73.00	R$ 83.00				
1	3	R$ 73.00	R$ 234.00	1	1	R$ 2,320.93	R$ 8,594.54
					1		R$ 5,531.31
1	1	R$ 73.00	R$ 78.00		2		R$ 3,265.19
1	1	R$ 73.00	R$ 78.00		1		R$ 3,860.86
3	1	R$ 219.00	R$ 73.00	1	2	R$ 2,734.67	R$ 9,226.48
	4		R$ 307.00		1		R$ 4,891.45
					1		R$ 4,456.26
	2		R$ 151.00	1	2	R$ 2,185.46	R$ 7,296.86
					1		R$ 3,159.49
	2		R$ 156.00		1		R$ 3,057.06
	2		R$ 166.00		1		R$ 3,625.71
1	3	R$ 73.00	R$ 249.00		1		R$ 3,351.04
2		R$ 146.00			2		R$ 5,739.38
1	2	R$ 73.00	R$ 161.00		1		R$ 2,277.04
	2		R$ 156.00				
					1		R$ 2,929.66
1		R$ 73.00			1		R$ 430.32
	1		R$ 78.00				
4	3	R$ 292.00	R$ 234.00				
					2		R$ 2,646.50
5	2	R$ 365.00	R$ 156.00	1	1	R$ 1,184.90	R$ 1,494.65
	1		R$ 73.00				
	2		R$ 156.00				
	2		R$ 146.00				
	1		R$ 83.00				
	2		R$ 166.00				
1	1	R$ 73.00	R$ 78.00				
1	2	R$ 78.00	R$ 161.00				
1	1	R$ 73.00	R$ 83.00				
	4		R$ 292.00				

1		R$ 73.00					
	1		R$ 78.00				
	1		R$ 73.00				
					1		R$ 1,296.87
1	2	R$ 78.00	R$ 136.00				
	1		R$ 78.00				
	1		R$ 73.00				
				1	1	R$ 4,116.58	R$ 2,902.96
3	3	R$ 224.00	R$ 249.00				
	1		R$ 83.00				
2	1	R$ 146.00	R$ 78.00	1	2	R$ 1,116.04	R$ 3,006.47
1		R$ 73.00					
	2		R$ 156.00				
	2		R$ 151.00				
	1		R$ 78.00				
	2		R$ 166.00				
				1	1	R$ 3,997.87	R$ 4,594.10
1	1	R$ 75.40	R$ 68.00				
1	2	R$ 73.00	R$ 151.00				
4	3	R$ 302.00	R$ 244.00		1		R$ 2,917.64
1		R$ 78.00					
1	3	R$ 73.00	R$ 229.00				
	1		R$ 78.00				
1		R$ 73.00					
1	2	R$ 66.00	R$ 136.00				
	1		R$ 73.00				
1	1	R$ 73.00	R$ 78.00				
1		R$ 73.00					

1	1	R$ 78.00	R$ 83.00				
	1		R$ 83.00				
1		R$ 78.00					
				1		R$ 160.32	
				1	1	R$ 2,252.16	R$ 2,203.00
	3		R$ 234.00				
3	1	R$ 219.00	R$ 78.00				
1		R$ 73.00					
2		R$ 146.00					
1		R$ 73.00					
2		R$ 146.00					
1		R$ 73.00					
	1		R$ 78.00				
1	1	R$ 73.00	R$ 68.00				
				1		R$ 2,091.15	
2		R$ 146.00					
2		R$ 146.00					
1	2	R$ 73.00	R$ 146.00				
	1		R$ 83.00				
3	1	R$ 219.00	R$ 83.00				
1		R$ 73.00		1		R$ 1,238.67	
	1		R$ 78.00				
1		R$ 73.00		1		R$ 1,805.65	
1		R$ 73.00		1	1	R$ 22,306.25	R$ 15,011.89
				3	2	R$ 6,797.76	R$ 4,879.48
2		R$ 146.00		1		R$ 1,619.02	
1		R$ 73.00		1		R$ 2,586.09	
2	1	R$ 146.00	R$ 78.00				
1		R$ 73.00					

1		R$ 73.00					
				2		R$ 5,406.59	
1		R$ 73.00		1		R$ 3,331.19	
2		R$ 146.00		1		R$ 2,504.46	
5		R$ 365.00		2	2	R$ 15,257.55	R$ 11,620.13
5		R$ 370.00		3		R$ 4,560.36	
6	2	R$ 438.00	R$ 166.00	1		R$ 5,277.59	
1	1	R$ 73.00	R$ 83.00	1	2	R$ 11,724.72	R$ 8,235.67
	1		R$ 83.00	1		R$ 6,765.06	
				1		R$ 2,542.86	
1	4	R$ 73.00	R$ 327.00	1		R$ 3,133.05	
				1		R$ 5,140.40	
	1		R$ 83.00	2		R$ 5,829.48	
				2		R$ 5,481.46	
2	2	R$ 154.90	R$ 156.00	1		R$ 2,250.17	
2		R$ 146.00		2		R$ 5,423.01	
				1		R$ 3,610.32	
				1		R$ 5,439.47	
1		R$ 73.00		1		R$ 5,812.17	
1	1	R$ 73.00	R$ 73.00	1		R$ 4,391.09	
1		R$ 73.00		1		R$ 5,821.54	
2		R$ 146.00		2		R$ 5,366.84	
1		R$ 73.00		2		R$ 5,952.75	
2	1	R$ 146.00	R$ 78.00	1		R$ 6,988.80	
1		R$ 73.00		1		R$ 5,773.06	
				1	3	R$ 12,419.11	R$ 4,855.41
3		R$ 219.00		1		R$ 8,343.80	
	1		R$ 78.00	2	1	R$ 13,831.85	R$ 4,109.16
1		R$ 73.00		1		R$ 10,042.50	
4	1	R$ 292.00	R$ 83.00	1		R$ 10,374.41	
1		R$ 73.00		2		R$ 10,908.10	
1		R$ 73.00		3	1	R$ 13,134.78	R$ 2,971.72
2	1	R$ 146.00	R$ 78.00	3		R$ 10,755.25	
1		R$ 73.00		3	1	R$ 19,215.53	R$ 6,646.35
4		R$ 292.00		6	4	R$ 22,056.66	R$ 9,275.14
				1	2	R$ 17,091.89	R$ 3,842.22
	1		R$ 78.00	1		R$ 14,826.32	
1		R$ 73.00		1		R$ 15,020.68	
1		R$ 88.40		1		R$ 10,947.27	
				2		R$ 16,753.62	
				1		R$ 11,980.94	
6		R$ 438.00		6	1	R$ 19,599.64	R$ 2,605.09
				1		R$ 20,585.25	
				1		R$ 24,609.33	
2		R$ 131.00		2		R$ 23,830.67	
2		R$ 151.00		1		R$ 25,440.97	
3		R$ 219.00		1		R$ 24,975.85	
1		R$ 73.00		2		R$ 29,577.36	
5	4	R$ 365.00	R$ 307.00	4	1	R$ 43,303.82	R$ 11,997.59
3		R$ 219.00		4		R$ 33,711.15	
3	1	R$ 219.00	R$ 78.00	1		R$ 32,192.99	
2	2	R$ 146.00	R$ 156.00	2		R$ 38,812.66	
1		R$ 73.00		2		R$ 39,020.49	

				1		R$ 37,950.43	
7		R$ 511.00		8	1	R$ 60,106.51	R$ 5,485.14

CUSTO ASSISTENCIAL				
Classificação	Beneficiários avaliados	Custo anterior (R$)	Custo posterior (R$)	Diferença (R$)
Em risco de fragilização	85	349,926.23	334,872.50	15,053.73
Idoso frágil	25	309,559.98	208,501.33	101,058.65
Idoso Robusto	113	486,114.47	325,356.06	160,758.41
**TOTAL**	**223**	**1,145,600.68**	**868,729.89**	**276,870.79**

INTERNAÇ				
Classificação	Internações hospitalares anteriores	Internações hospitalares posteriores	Diferença Nº Internações	Variação Nº de I
Em risco de fragilização	36	30	6	Redução de
Idoso frágil	44	22	22	Redução de
Idoso Robusto	47	27	20	Redução de
**TOTAL**	**127**	**79**	**48**	**Redução de**

CONSULTAS I				
Classificação	Consultas em PA anteriores	Consultas em PA posteriores	Diferença Nº Consultas PA	Variação Nº de C
Em risco de fragilização	59	54	5	Redução de
Idoso frágil	47	15	32	Redução de
Idoso Robusto	72	70	2	Redução de
**TOTAL**	**178**	**139**	**39**	**Redução de**

CONSULTAS ELETIVAS (S				
Classificação	Consultas Eletivas anteriores	Consultas Eletivas posteriores	Diferença Nº Consultas Eletivas	Variação Nº de Eletiva
Em risco de fragilização	263	246	17	Redução de
Idoso frágil	65	31	34	Redução de
Idoso Robusto	288	323	35	Aumento de
**TOTAL**	**616**	**600**	**16**	**Redução de**

CONSULTAS NO USIFAMI

Classificação	Consultas APS anteriores	Consultas APS posteriores	Diferença Nº Consultas APS	Variação Nº de Co
Em risco de fragilização	0	257	257	#DIV/0!
Idoso frágil	0	90	90	#DIV/0!
Idoso Robusto	0	251	251	#DIV/0!
**TOTAL**	**0**	**598**	**598**	**#DIV/0!**

EXAMES AMBUL				
Classificação	Exames ambulatoriais anteriores	Exames ambulatoriais posteriores	Diferença Nº Exames	Variação Nº d
Em risco de fragilização	1,304	1,769	465	Aumento de
Idoso frágil	332	351	19	Aumento de
Idoso Robusto	1,566	2,159	593	Aumento de
**TOTAL**	**3,202**	**4,279**	**1,077**	**Aumento de**

Variação Custo	
Redução de	-4.3%
Redução de	-32.6%
Redução de	-33.1%
Redução de	-24.2%

ÕES

nternações	Custo anterior (R$)	Custo posterior (R$)	Diferença Custo (R$)	Variação Custo Total das Internações	
-16.7%	243,256.52	207,200.61	36,055.91	Redução de	-14.8%
-50.0%	291,315.02	186,435.78	104,879.24	Redução de	-36.0%
-42.6%	373,863.90	187,097.73	186,766.17	Redução de	-50.0%
**-37.8%**	**908,435.44**	**580,734.12**	**327,701.32**	**Redução de**	**-36.1%**

EM PA

onsultas PA	Custo anterior (R$)	Custo posterior (R$)	Diferença Custo (R$)	Variação Custo Total	
-8.5%	4,320.90	4,207.00	113.90	Redução de	-2.6%
-68.1%	3,436.00	1,165.00	2,271.00	Redução de	-66.1%
-2.8%	5,303.80	5,470.00	(166.20)	Aumento de	3.1%
**-21.9%**	**13,060.70**	**10,842.00**	**2,218.70**	**Redução de**	**-17.0%**

SEM USIFAMILIA)

Consultas as	Custo anterior (R$)	Custo posterior (R$)	Diferença Custo (R$)	Variação Custo Total	
-6.5%	16,008.56	16,216.92	(208.36)	Aumento de	1.3%
-52.3%	3,751.68	2,013.00	1,738.68	Redução de	-46.3%
12.2%	17,065.86	20,832.12	(3,766.26)	Aumento de	22.1%
**-2.6%**	**36,826.10**	**39,062.04**	**(2,235.94)**	**Aumento de**	**6.1%**

LIA - CODIGO 8003

onsultas APS	Custo anterior (R$)	Custo posterior (R$)	Diferença Custo (R$)	Variação Custo Total	
**#DIV/0!**	-	2.57	(2.57)	**#DIV/0!**	**#DIV/0!**
**#DIV/0!**	-	0.90	(0.90)	**#DIV/0!**	**#DIV/0!**
**#DIV/0!**	-	2.51	(2.51)	**#DIV/0!**	**#DIV/0!**
**#DIV/0!**	**-**	**5.98**	**(5.98)**	**#DIV/0!**	**#DIV/0!**

ATORIAIS

e Exames	Custo anterior (R$)	Custo posterior (R$)	Diferença Custo (R$)	Variação Custo Total	
**35.7%**	48,298.11	60,263.29	(11,965.18)	**Aumento de**	**24.8%**
**5.7%**	8,823.46	8,288.52	534.94	**Redução de**	**-6.1%**
**37.9%**	63,782.40	76,973.79	(13,191.39)	**Aumento de**	**20.7%**
**33.6%**	**120,903.97**	**145,525.60**	**(24,621.63)**	**Aumento de**	**20.4%**
